# *Lactobacillus plantarum* CCFM405 against Rotenone-Induced Parkinson’s Disease Mice via Regulating Gut Microbiota and Branched-Chain Amino Acids Biosynthesis

**DOI:** 10.3390/nu15071737

**Published:** 2023-04-01

**Authors:** Chuanqi Chu, Leilei Yu, Yiwen Li, Hang Guo, Qixiao Zhai, Wei Chen, Fengwei Tian

**Affiliations:** 1 State Key Laboratory of Food Science and Technology, Jiangnan University, Wuxi 214122, China; 2 School of Food Science and Technology, Jiangnan University, Wuxi 214122, China; 3 Department of Food Science and Technology, The University of Georgia, Athens, GA 30602, USA; 4 National Engineering Research Center for Functional Food, Jiangnan University, Wuxi 214122, China

**Keywords:** Parkinson’s disease, gut–brain axis, *Lactobacillus plantarum*, branched amino acid

## Abstract

Recent studies have demonstrated that disturbances in the gut microbiota and microbiota -derived metabolites contribute to the pathogenesis of Parkinson’s disease (PD), suggesting that probiotic treatments that restore them may delay disease progression. This study aimed to examine the attenuating efficacy of *L. plantarum* CCFM405 and the potential mechanisms in mice with rotenone-induced PD. Our results indicate that *L. plantarum* CCFM405 ameliorated rotenone-induced motor deficits and constipation, decreased dopaminergic neuronal death, reduced intestinal inflammation and neuroinflammation, and raised dopamine levels, 5-HT, and associated metabolites in the striatal region of the brain in mice with PD. Sequencing of 16S rRNA from fecal microbiota revealed that *L. plantarum* CCFM405 normalized the gut bacterial composition in mice with PD, as evidenced by the increased relative abundance of the following genus, *Bifidobacterium*, *Turicibacter*, and *Faecalibaculum*, and decreased relative abundance of *Alistipes*, *Bilophila*, *Akkermansia*, and *Escherichia-Shigella*. The PICRUSt-predicted gut microbiota function revealed that *L. plantarum* CCFM405 enhanced the biosynthesis of amino acid pathways, particularly valine, leucine, and isoleucine (branched-chain amino acids, BCAAs). A non-metabolomic analysis of the serum and feces showed that *L. plantarum* CCFM405 markedly increased the levels of BCAAs. Pathway enrichment analysis based on the KEGG database further suggested that *L. plantarum* CCFM405 supplementation can promote BCAAs biosynthesis. Collectively, *L. plantarum* CCFM405 can help to prevent rotenone-induced PD by modulating the gut microbiota–metabolite axis. BCAAs may play a dominant role in *L. plantarum* CCFM405-associated neuroprotection in PD mice. This probiotic could be utilized as a potential food supplement in the management of PD.

## 1. Introduction

Parkinson’s disease (PD) is the second most prevalent neurodegenerative disorder of the central nervous system [1]. The global prevalence of PD is <0.5% in persons under 50 years of age and grows to 4% in those over 80 [2], causing a heavy burden on families and society. The substantia nigra (SN) and striatum are the primary areas of pathogenesis in the brains of patients with PD, with the death and reduction in dopaminergic neurons in the compact part of the SN being the direct cause of the reduction of dopamine (DA) projected into the striatum [3]. This results in resting tremor, bradykinesia, myotonia, and aberrant posture and gait [4]. The pathogenic mechanisms of PD may be related to abnormal accumulation of alpha-synuclein (α-syn). Neuroinflammation, apoptosis, oxidative stress, cellular stress responses, endoplasmic reticulum stress, neurotoxicity, and autophagy may contribute to the death of DA neurons and the frequent occurrence and progression of PD [3,5,6,7,8,9]. Studies have demonstrated that gastrointestinal symptoms such as constipation and inflammatory bowel disease often precede movement disorders in people with PD [10] and may accelerate motor dysfunction, indicating that PD may begin in the gastrointestinal tract. The “gut-originality” is a critical issue in the current research on the pathogenesis of PD.

The gut microbiota plays an important role in central nervous system function. It regulates brain function and behavioral performance through multiple pathways, including neural (vagus, enteric nervous system, and spinal nerves), immune (cytokines), endocrine pathways (cortisol), and microbiota-driven metabolites, constituting the microbiota–gut–brain axis (MGBA) [11,12]. The imbalance of the gut microbiota may be associated with the development and clinical characteristics of PD [13]. Changes in intestinal bacteria metabolites, abnormal intestinal function, and intestinal inflammation may also result from an imbalance in gut bacteria [14,15]. The largest meta-analysis of gut microbes from PD patients to date included 2269 16S rRNA gene samples and 236 samples by shotgun metagenomics. The results showed that the relative abundances of *Roseburia*, *Faecalibacterium*, *Blautia*, *Lachnospira*, and *Prevotella* were significantly decreased, while the relative abundances of *Streptococcus*, *Bifidobacterium*, *Akkermansia*, and *Desulfovibrio* were significantly increased. Notably, the relative abundance of potentially pro-inflammatory bacteria, genes, and pathways in PD patients was significantly increased, while potentially anti-inflammatory bacteria, genes, and pathways were significantly decreased. This may result in a decrease in potentially anti-inflammatory substances (short-chain fatty acids, SCFAs) and an increase in potentially pro-inflammatory substances (lipopolysaccharides, hydrogen sulfide, and glutamate) [16].

In a rotenone-induced PD mouse model, Zhao et al. reported that fecal microbiota transplantation (FMT) protected mice from weight loss, motor deficits, and gastrointestinal impairment. Simultaneously, it lowered lipopolysaccharide (LPS) concentrations in the blood, colon, and SN, inhibiting TLR4/MyD88/NF-kB signal pathway activation, and downmodulating pro-inflammatory protein expression in the SN and colon [17], similar to the findings of another study [18]. These findings highlight the importance of the gut microbiota in the pathophysiology of PD and demonstrate that modulating the microbiota compositions may attenuate motor and non-motor abnormalities. Therefore, the microbiota may be an intervention target for PD therapy and management.

Targeting the abnormal gut microbiota compositions of patients with PD and their pro-inflammatory characteristics has generated research interest in modifying the gut microbiota, such as through the use of probiotics, as a potentially novel method for managing the disease. *Lactobacillus plantarum* PS128 (PS128) is a novel psychobiotic strain that alleviated tic-like behaviors or MPTP-induced motor dysfunction by modulating the MGBA [19,20]. Notably, PS128 supplementation with constant anti-Parkinsonian medication was found to improve the Unified Parkinson’s Disease Rating Scale (UPDRS) motor score and quality of life of patients with PD [21]. Similarly, *Lactobacillus plantarum* DP189 was reported to delay MPTP-induced neurodegeneration by reducing accumulation of α-syn and oxidative stress in the SN, inhibiting neuroinflammation and reshaping the composition of gut microbes [22]. In a rotenone-induced PD model, a probiotic cocktail containing *Lactobacillus rhamnosus* GG, *Bifidobacterium animalis lactis*, and *Lactobacillus acidophilus* attenuated neuronal loss in the nigrostriatal pathway, partially by increasing the butyrate level [23]. Rotenone-induced PD animal models have shown great potential for studying PD pathology, motor and non-motor symptoms, gene–environment interactions, and the role of the gut–brain axis in the pathogenesis of PD [24,25,26,27].

*Lactobacillus plantarum* CCFM405 (*L. plantarum* CCFM405), isolated from pickle samples from Sichuan Province (China), was reported to have strong antioxidant capacity, with a hydroxyl radical scavenging rate of 32.02% [28]. Here, we aimed to investigate whether *L. plantarum* CCFM405 has neuroprotective properties in mice with rotenone-induced PD. Additionally, we analyzed neurodegeneration-associated influences in dopamine neuroprojection areas to better understand their mechanisms. We also examined the compositions and functional prediction of the gut microbiota using 16S rRNA sequencing and compositions of the fecal and serum metabolites using non-target metabolomics to further evaluate the significance of the gut microbiota–metabolite–brain axis.

## 2. Materials and Methods

### 2.1. Animals

In this experiment, 7-week-old C57BL/6J strain mice weighing (20 ± 2) g were purchased from the Beijing Vital River Laboratory Animal Technology Co., Ltd. (Jiaxing, China). The mice were acclimatized and fed for one week before treatment. Mice were placed in a pathogen-free environment (12 h light/dark cycle) at constant temperature (24 ± 2.0 °C) and relative humidity (55 ± 10%). They also had unrestricted access to water and normal rodent food. The Animal Ethics Committee of Jiangnan University approved all the experimental protocols. The ethics number is JN. No. 20201115c1320120 [303].

### 2.2. Experimental Design and Rotenone Treatment

**Figure 1:**
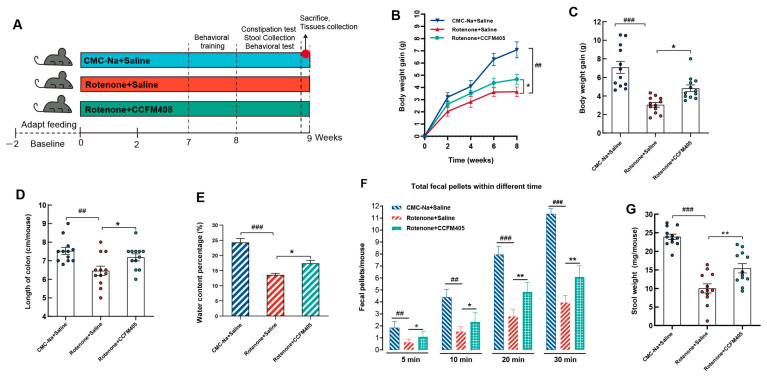
Effects of *L. plantarum* CCFM405 on body weight and gastrointestinal function in rotenone-induced PD mice. (**A**) Timeline of experimental design; (**B**) changes in body weight during feeding; (**C**) weight gain at the endpoint; (**D**) colon length of mice; (**E**) fecal water content of mice at the intervention endpoint; (**F**) number of mice defecation grains at different periods; (**G**) fecal weight of mice at the intervention endpoint. *n* = 12/group. Data are means with SEM. ## *p* < 0.01, ### *p* < 0.001 signs Rotenone+Saline group vs. CMC-Na+Saline group; * *p* < 0.05, ** *p* < 0.01 signs Rotenone+CCFM405 group vs. Rotenone+Saline group.

Figure 1A depicts the grouping and schedule for the animal experiments. Rotenone (30 mg/kg) is known to destroy the functional gastrointestinal system and the dopaminergic nigrostriatal pathway and to induce PD symptoms [24,25]. According to a previous protocol, a PD-like mouse model was successfully established by chronic oral administration of Rotenone (suspended in 0.5% percent carboxymethyl cellulose sodium salt (CMC-Na) with Tween-20) into mice for eight weeks [24] (Figure 1A). After one week of acclimatization feeding, 8-week-old C57BL/6J mice were randomly divided into three groups (12 mice per group): (1) the CMC-Na+Saline group, which was given a CMC-Na and normal saline once daily by gavage; (2) the Rotenone+Saline group, which was given a Rotenone (30 mg/kg) and normal saline once daily by gavage; and (3) the Rotenone+*L. plantarum* CCFM405 group, which was given a Rotenone (30 mg/kg) and *L. plantarum* CCFM405 (10^9^ CFU/200 μL saline) once daily by gavage. To reduce animal suffering, all experimental procedures were performed under anesthetic. After sacrifice, the whole brain, striatum, colonic tissue, serum, and feces were taken (Figure 1A).

### 2.3. Measurement of Colon Motility and Stool Collection

The procedure is based on a previous study with appropriate adjustments [25]. After the last rotenone treatment, the frequency of bowel movements every 30 min was recorded in these three groups. The testing period ran from 9:00 a.m. through 11:00 a.m. Each animal was taken from its cage and put without food or drink for 30 min in a clean, sterilized plastic cage. Immediately after ejection, the feces were collected and placed in 1.5 mL sterile EP tubes. The frequency of defecation was calculated for each mouse based on the weight of the stool, the number of fecal pellets, and the water content percentage for quantitative analysis.

### 2.4. Behavioral Tests for Motor Functions

The motor function of mice was evaluated by the pole test, the beam walking test, the rota-rod test, and the open field test [20,29,30]. For detailed behavioral experimental protocols, please refer to Supplementary methods in Supplementary Information. Before conducting the behavioral tests, mice performed daily behavioral training for three consecutive days (Figure 1A) to reduce the mistakes occurring during the formal testing phase, owing to a lack of familiarity with the testing instrument. Behavioral assessment was administered in the eighth week (Figure 1A).

### 2.5. Measurement of Neurotransmitters

High-performance liquid chromatography with a fluorescence detector (Waters 2475, Milford, MA, USA) was used to measure the levels of striatal dopamine (DA, #PHR1090, Sigma-Aldrich Co., Ltd., St. Louis, MO, USA), 5-hydroxytryptamine (5-HT, Sigma Aldrich, #14927), and their metabolites, including 3, 4-dihydroxyphenylacetic (DOPAC, Sigma Aldrich, #11569), homovanillic acid (HVA, Sigma Aldrich, #69673), and 5-hydroxyindoleacetic acid (5-HIAA, Sigma Aldrich, #55697), following the same procedures as before with minor adjustments [29]. The separation system (Waters 2695) was used with an Atlan-tis T3 column (150 mm × 4.6 mm, 5 μm, Waters, Milford, MA, USA). The mobile phases were composed of water, acetonitrile, and 0.01 M PBS (pH = 4.0). Briefly, the striatum was homogenized in 0.2 M perchloric acid (10 μL/mg of striatum tissue) by sonication and the homogenate was centrifuged at 13,000× *g* 4 °C for 10 min. Then, the supernatants were collected and filtered through a 0.22-μm filter and 25 μL of sample was injected into the column. The linearity ranges of neurotransmitters were determined by serial concentrations of a standard solution before detection.

### 2.6. Immunohistochemistry and Image Analysis

For immunochemistry, mice were thoroughly anesthetized with isoflurane and perfused transcardially with ice-cold PBS, followed by 4% paraformaldehyde in 0.01 M PBS at a pH of 7.4. Overnight, tissues were fixed in 4% paraformaldehyde at 4 °C. The brains were dissected, fixed, dried, paraffin-embedded, and sliced to a thickness of 4 m. Every fifth segment containing the SN was taken, and three sections were immunostained in each sample. After inhibiting endogenous peroxidase activity with 3% H_2_O_2_ and antigen retrieval using citric acid buffer, the sections were blocked with 5% goat serum and treated overnight at 4 °C with a primary antibody against TH (MAB318, Millipore, 1:400, Merck KGaA, Germany), GFAP (ab68428, Abcam, 1:500, Cambridge, MA, USA), Iba-1 (ab178846, Abcam, 1:2000, Cambridge, MA, USA) for brains. On the second day, the sections were incubated with a biotin-conjugated goat anti-rabbit IgG antibody (1:200, GB23303, Servicebio, Wuhan, China), and the avidin–biotin complex (ABC) system and nickel-enhanced diaminobenzidine (DAB) incubation were used to stain the sections. A double-port inverted microscope was used to capture images (ECLIPSE Ti-S, Nikon, Tokyo, Japan). Image-Pro Plus version 6.0 software (Media Cybernetics, Inc., Rockville, MD, USA) was utilized to determine the area and density of the dyed zone, as well as the integrated optical density (IOD) of the IHC section. The mean densitometry of the digital picture was identified as the representative TH staining intensity (representing the relative TH expression level). The signal density of tissue portions from five randomly selected fields was counted and statistically analyzed in a blinded manner.

### 2.7. Gut Microbiota Profiling and Functional Prediction Analysis

Gut microbiota structure analyses were conducted using the MicrobiomeAnalyst Platform [31,32]. The number of operational taxonomic units (OTUs) shared and unique genera among the three groups was completed using a Venn diagram (https://www.bioincloud.tech (accessed on 20 January 2023)). Alpha diversity was calculated using the chao1 and observed index with Nonparametric Mann–Whitney tests. For the beta diversity and significant testing, the Interactive PCoA 3D method was used with the Jaccard Index at the genus level with permutational multivariate analysis of variance (PERMANOVA) applied as the statistical method. The linear discriminant analysis (LDA) and random forest (RF) methods were used for the biomarker analysis. The platform performs a non-parametric factorial Kruskal–Wallis sum-rank test to identify features with significant differential abundance considering the class of interest, followed by LDA to calculate the effect size of each differentially abundant feature. The features are considered significant depending on their adjusted *p*-value. The default adj. *p*-value cutoff = 0.05 and the LDA score is 3.0. The Metagenome Seq method was also used to evaluate differential abundance in sparse marker gene survey data using a zero-inflated Gaussian (ZIG) fit model to account for under sampling and sparsity in OTU count data after normalizing the data through cumulative sum scaling (CSS) [33].

Phylogenetic Investigation of Communities by Reconstruction of Unobserved States (PICRUSt2) [34] was used to predict the metagenome to evaluate the functions of gut microbiota among the three groups. An OTU table was used for predicting the metagenome based on the Kyoto Encyclopedia of Genes and Genomes (KEGG) Orthology (KO) annotations. Metabolic module enrichment analysis was completed using the Wekemo Bioincloud (https://www.bioincloud.tech (accessed on 20 January 2023)).

### 2.8. Metabolomic Data Analysis

The linearity ranges of neurotransmitters were determined by serial concentrations of standard solution before detection. Mass raw data files were imported into Compound Discovery 3.3 (Thermo Fisher Scientific, Waltham, MA, USA) where peaks were automatically processed using an untargeted metabolic workflow. Metabolites were identified by matching mzCloud and ChemSpider databases. For normalization, the spectral counts of each metabolite were divided by the total spectral counts of all metabolites from the same sample. The data transformation is log transformation (base 10). Pareto scaling is used to perform data scaling. Based on the relative quantification of metabolites, partial least squares discriminant analysis (PLS-DA) of all samples and the Pearson correlation coefficient between QC samples were set as standards in the assessment of stability in the metabolomic data sets. The number of permutation tests was set to 200. Differential metabolites amongst the three groups were identified based on one-way ANOVA with Tukey’s Honest Significant Difference (HSD) post hoc Tests (Adjusted *p*-value < 0.05). Hierarchical cluster analysis, pathway analysis, and metabolite set enrichment analysis were performed in MetaboAnalyst 5.0 (https://www.metaboanalyst.ca (accessed on 10 February 2023)).

### 2.9. Sample Collection and Tissue Preparation

After behavioral testing, the mice were thoroughly anesthetized with isoflurane, then infused with cold-sterile saline, and fresh striatal and midbrain tissue was removed. The tissues were instantly flash-frozen in liquid nitrogen and kept at −80 °C. Blood samples from the orbits were centrifuged at 3000 rpm for 15 min to extract serum, which was then frozen at −80 °C for further non-target metabolomic analyses. Other detailed experimental methods and procedures, including quantitative real-time polymerase chain reaction (qRT-PCR), AB-PAS and HE staining of the colon, enzyme-linked immunosorbent assay (Elisa), metagenomic sequencing, preparation of fecal and serum samples for untargeted metabolomics analysis, liquid chromatography (LC), and mass spectrometry (MS) system and parameter settings can be seen in the Supplementary Information.

### 2.10. Statistical Analysis

Statistical analysis regarding the gut microbiota and non-target metabolome are shown in the Supplementary Information. GraphPad Prism version 8.3 (GraphPad Inc., La Jolla, CA, USA) was used for data analysis and visualization. The data were tested for normality using the Shapiro–Wilk method and Q-Q plots before the data were analyzed for significance. For data that conformed to a normal distribution, unpaired Student’s t-test was performed to analyze significant differences between the two groups; For data that did not conform to a normal distribution, the Mann–Whitney test was used to analyze significant differences between the two groups. FDR (Benjamini–Hochberg method) was used for high dimensional data, so *p*-values were corrected. Data are presented as mean ± SEM (standard error of the mean), and *p*  <  0.05 was set as the threshold for significance.

## 3. Results

### 3.1. Effects of L. plantarum CCFM405 on Body Weight and Gastrointestinal Function in Rotenone-Induced PD Mice

Rotenone induces movement disorders and gastrointestinal dysfunction in stable animal models of PD [24,25]. According to the experimental protocol in Figure 1A, after daily gavage of rotenone for 2 h, *L. plantarum* CCFM405 supplementation was given to mice in the intervention group. Training on the pole, balance beam, and rota-rod tests was performed on the seventh week, and formal behavioral and gastrointestinal function tests were performed on the eighth week. As shown in Figure 1B, from the second week, the body weight of the mice in the Rotenone+Saline group started to decrease compared to that of the CMC-Na+Saline group. In contrast, the body weight of mice in the *L. plantarum* CCFM405 group markedly increased compared to that of the mice in the Rotenone+Saline group. By week eight, the body weight of mice in the Rotenone+Saline group was markedly lower compared to that of the CMC-Na+Saline group (*p* < 0.01), and the body weight of mice in the Rotenone+CCFM405 group was dramatically higher compared to that of the model group (*p* < 0.05). This indicated that *L. plantarum* CCFM405 had a positive inhibitory effect on the decrease in body weight of mice caused by rotenone (Figure 1C).

The intake of rotenone caused gastrointestinal dysfunction in mice. The most obvious symptom was constipation [25]. PD is a motor disorder caused by the loss of dopaminergic neurons in the SN, but gastrointestinal dysfunction is also a common symptom. There is evidence that these gastrointestinal problems may exist long before PD is diagnosed [35], which suggests that the disease may originate in the gut. Therefore, we assessed the constipation of mice by examining the number and weight of fecal pellets defecated at different periods, and the water content of feces. As shown in Figure 1F, the intake of rotenone resulted in a significant decrease in the number of fecal pellets compared to that in the control group (*p* < 0.01), while the supplementation of *L. plantarum* CCFM405 markedly increased the number of fecal pellets in PD mice (*p* < 0.05). For fecal weight and water content, *L. plantarum* CCFM405 also markedly reversed the adverse effects caused by rotenone (Figure 1E,G). In addition, by measuring the colon length of mice after euthanasia, we found that rotenone markedly shortened the colon length. In contrast, the colon length of mice in the Rotenone+CCFM405 group was increased significantly compared to that of the Rotenone+Saline group (Figure 1D). Therefore, *L. plantarum* CCFM405 could effectively alleviate constipation symptoms, and colon shortening in PD mice.

### 3.2. Effects of L. plantarum CCFM405 on Motor Function in Rotenone-Induced PD Mice

**Figure 2:**
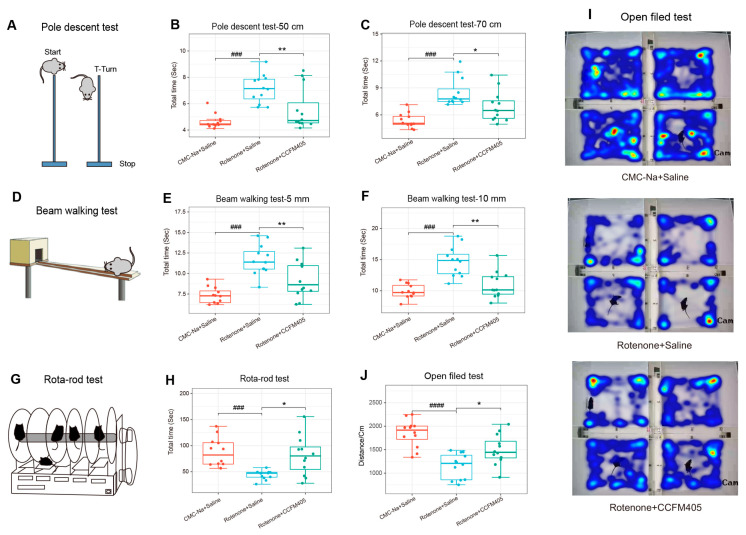
Effect of *L. plantarum* CCFM405 on movement performance in rotenone-challenged PD mice. (**A**) Cartoon diagram of the Pole descent test; (**B**) Pole descent test (50 cm pole); (**C**) Pole descent test (70 cm pole); (**D**) cartoon diagram of the Balance walking test; (**E**) Balance walking test (5 mm wide balance beam); (**F**) Balance walking test (10 mm wide balance beam); (**G**) cartoon diagram of the Rotating rod test; (**H**) Rotating rod test (30 rpm); (**I**) representative heat maps of tracking movement trajectories of mice in each group; (**J**) Open Field test. *n* = 11–12/group. Data are means with SEM. ### *p* < 0.001, #### *p* < 0.0001 signs Rotenone+Saline group vs. CMC-Na+Saline group; * *p* < 0.05, ** *p* < 0.01 signs Rotenone+CCFM405 group vs. Rotenone+Saline grup.

The pole test, the beam walking test, rota-rod test, and the open field tests are classic behavioral experiments to evaluate motor function in animal models with PD. As shown in Figure 2A–C, we used two climbing poles with different heights to assess the motor function in mice exposed to rotenone. On the 50 cm climbing pole, treatment with rotenone markedly increased the time required to climb from the top to the bottom of the pole compared to that in the control group (*p* < 0.01). In contrast, the intake of *L. plantarum* CCFM405 markedly reduced the total time required to climb the pole (*p* < 0.01), similar to the results obtained for 70 cm pole climbing. We further examined the coordination of PD mice by using the beam walking test (Figure 2D–F). Treatment with rotenone markedly increased the total time required to crawl, and involuntary body shaking was observed in PD mice during the test. Supplementation with *L. plantarum* CCFM405 markedly reduced the time required to walk the balance beam. We further used the rotating rod test to detect fine coordination of PD mice. The results showed that the rotation time on the rotating stick apparatus in the Rotenone+Saline group was markedly reduced compared with that of the CMC-Na+Saline group (*p* < 0.001), and *L. plantarum* CCFM405 markedly restored their stay time on the rotating rod (*p* < 0.05) (Figure 2G,H). Similarly, as shown in Figure 2I,J, *L. plantarum* CCFM405 markedly increased the total distance traveled in the open field test compared to that in the model group mice (*p* < 0.05). Overall, these four behavioral tests indicated that *L. plantarum* CCFM405 could effectively alleviate motor impairment in rotenone-induced PD mice.

### 3.3. Effects of L. plantarum CCFM405 on Dopaminergic Neurons and Neurotransmitters in the Striatum of Rotenone-Induced PD Mice

**Figure 3:**
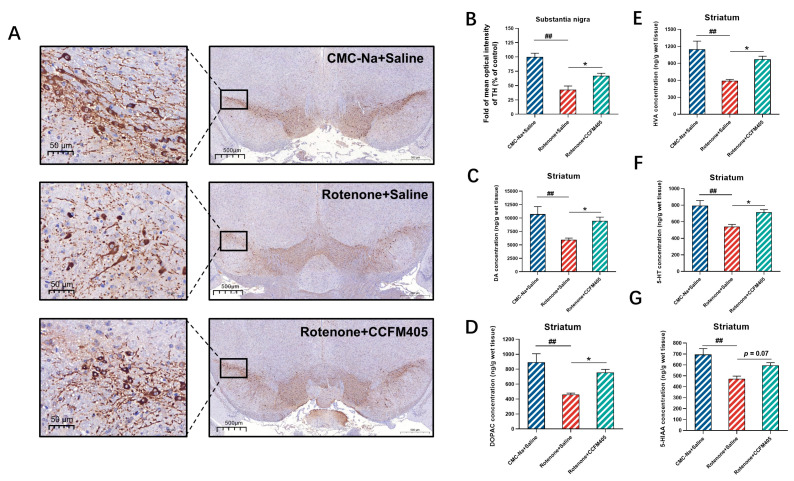
Effects of *L. plantarum* CCFM405 on dopaminergic neurons and neurotransmitters in the striatum of rotenone-challenged PD mice. (**A**) Representative images of TH immunohistochemistry in the left and right substantia nigra. Scale (left: 50 μm right: 500 μm) (*n* = 3/group); (**B**) quantitative evaluation of average optical density (OD) of TH positive area in the left and right substantia nigra (*n* = 3/group); The concentrations of neurotransmitters in the unilateral striatu (**C**) DA, dopamine; (**D**) DOPAC, 3,4-dihydroxyphenylacetic acid; (**E**) HVA, homovanillic acid; (**F**) 5-HT, 5-hydroxytryptamine; (**G**) 5-HIAA, 5-hydroxyindoleacetic acid. *n* = 8/group. Data are means with SEM. ## *p* < 0.01 signs Rotenone + Saline group vs. CMC-Na + Saline group; * *p* < 0.05 signs Rotenone+CCFM405 group vs. Rotenone+Saline grup.

Immunohistochemical (IHC) staining was used to assess the effect of *L. plantarum* CCFM405 on the survival of dopaminergic neurons in the substantia nigra and to determine whether tyrosine hydroxylase (TH, the rate-limiting enzyme for dopamine synthesis and used as a marker for dopaminergic neurons) expression correlated with changes in the dopamine levels. As shown in Figure 3A,B, the results of immunohistochemistry showed that the mean optical density values of TH were markedly lower in the model group of mice compared with that of the CMC-Na+Saline group (*p* < 0.001). Simultaneously, the supplementation of *L. plantarum* CCFM405 markedly increased TH expression in the substantia nigra (*p* < 0.05). We further used high-performance liquid chromatography (HPLC) to detect the levels of DA and 5-HT and their metabolites DOPAC, HVA, and 5-HIAA in the striatum of mice. As shown in Figure 3C, the striatal dopamine levels in the model group were markedly lower than those in the CMC-Na+Saline group (*p* < 0.001). In contrast, the striatal dopamine levels in the *L. plantarum* CCFM405-supplemented group were markedly higher than those in the model group (*p* < 0.05). Similarly, the levels of DOPAC and HVA in the striatum of mice in the CMC-Na+Saline group were markedly increased compared with those in the model group. Additionally, mice fed *L. plantarum* CCFM405 showed higher levels of DOPAC and HVA than mice in the model group (Figure 3D,G). Mice in the model group showed markedly lower levels of 5-HT and 5-HIAA compared to that in the control group mice (*p* < 0.001). In contrast, *L. plantarum* CCFM405 showed elevated levels of 5-HT and 5-HIAA, compared to that in the model mice (Figure 3E,F, *p* < 0.05, *p* = 0.07). These findings suggest that *L. plantarum* CCFM405 can affect striatal neurotransmitter metabolism by inhibiting rotenone-induced reductions in the levels of DA, 5-HT, and their related metabolites.

### 3.4. Effects of L. plantarum CCFM405 on Microglia and Astrocytes in the Nigra Region of the Brain of Rotenone-Induced PD Mice

**Figure 4:**
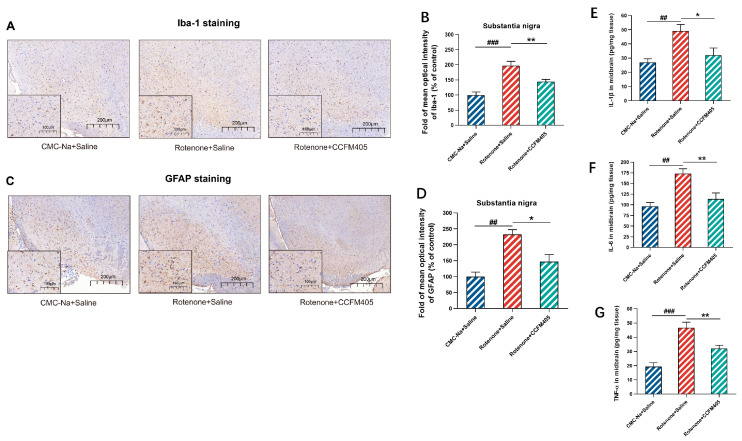
Effects of *L. plantarum* CCFM405 on microglia and astrocytes in the SN region of the brain in rotenone-challenged PD mice. (**A**) Representative images of Iba-1 immunohistochemistry in the SN of mouse brain; Scale: 200 μm. (**B**) Average optical density analysis of Iba-1 positive cells in the SN region of mouse brain (*n* = 3/group); (**C**) representative images of GFAP immunohistochemistry in the SN of mouse brain; Scale: 200 μm. (**D**) Average optical density analysis of GFAP positive cells in the SN of mouse brain; Scale: 200 μm (*n* = 3/group); (**E**–**G**) the concentration of IL-1β, IL-6 and TNF-α in the midbrain of PD mice (*n* = 8/group). Data are means with SEM. ## *p* < 0.01, ### *p* < 0.001 signs Rotenone+Saline group vs. CMC-Na+Saline group; * *p* < 0.05, ** *p* < 0.01, signs Rotenone+CCFM405 group vs. Rotenone+Saline grup.

Neuroinflammation is an immunological response triggered in the central nervous system by microglia and astrocytes, and multiple studies have implicated neuroinflammation in the onset and progression of PD. It activates microglia in the brain that then generates pro-inflammatory cytokines through TLR4 receptors [36]. We used immunohistochemical experiments to investigate the effect of *L. plantarum* CCFM405 on the degree of activation of glial cells in the substantia nigra region of the brain in rotenone-induced PD mice. As shown in Figure 4A,B, compared with the CMC-Na+Saline group, rotenone treatment markedly increased the expression of Iba-1 in the nigrostriatal area of PD mice (*p* < 0.001), whereas the expression of Iba-1 in the mice of the *L. plantarum* CCFM405 group was markedly lower compared to that in the model group (*p* < 0.01). Similarly, the results of glial fibrillary acidic protein (GFAP) immunohistochemistry showed that the mean optical density values of GFAP in the nigrostriatal region of mice in the model group were more than twice as high as those of mice in the CMC-Na+Saline group (*p* < 0.01). Simultaneously, the supplementation of *L. plantarum* CCFM405 markedly reduced the expression of GFAP in PD mice (*p* < 0.05) (Figure 4C,D). To further investigate whether glial cell activation is accompanied by neuroinflammation, we measured the concentrations of IL-1β, IL-6, and TNF-α in the midbrain using ELISA. As shown in Figure 4E–G, the levels of IL-1β, IL-6, and TNF-α in the midbrain of mice in the rotenone-treated group were markedly increased compared to those in the normal healthy group (*p* < 0.01, *p* < 0.001, and *p* < 0.001). The IL-1β, IL-6, and TNF-α levels in the midbrain of mice were markedly lower in the *L. plantarum* CCFM405 intervention group compared to those in the model group (*p* < 0.05, *p* < 0.01, and *p* < 0.01). The above results indicated that *L. plantarum* CCFM405 markedly inhibited the activation of microglia and astrocytes in the nigrostriatal region and reduced the levels of pro-inflammatory cytokines in the midbrain, ultimately inhibiting neuroinflammation in PD mice.

### 3.5. Effects of L. plantarum CCFM405 on Intestinal Inflammation and Goblet Cell Count in the Colon in Rotenone-Induced PD Mice

**Figure 5:**
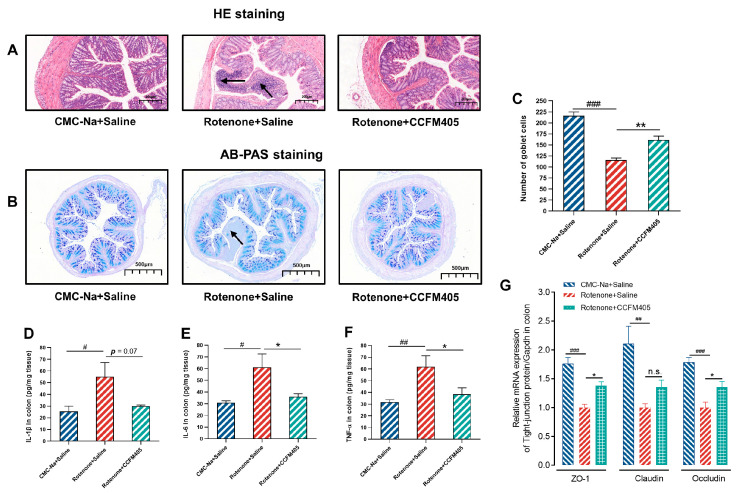
Effects of *L. plantarum* CCFM405 on intestinal inflammation and goblet cell count in the colon in rotenone-induced PD mice. (**A**) Representative images of H&E staining in the colon; the black arrows show inflammatory cell infiltration (*n* = 3/group); (**B**) representative images of AB-PAS staining of the colon (*n* = 5/group); (**C**) the number of goblet cells in the colon; (**D**–**F**) the concentration of IL-1β, IL-6, TNF-α in the colon (*n* = 8/group); (**G**) relative expression of colonic tight junction protein mRNA (*n* = 4/group). Data are means with SEM. # *p* < 0.05, ## *p* < 0.01, ### *p* < 0.001 signs Rotenone+Saline group vs. CMC-Na+Saline group; * *p* < 0.05, ** *p* < 0.01 signs Rotenone+CCFM405 group vs. Rotenone+Saline grup. “n.s.” means there was no noticeable difference between the groups.

A prospective cohort study of patients with colitis (over 12,000) and normal healthy individuals (nearly 6000) followed for an average of seven years showed that patients had a markedly higher risk of PD within three years of colitis diagnosis. In addition, a significant association was observed between microscopic colitis and the risk of developing PD [37]. Therefore, to investigate whether rotenone gavage causes colitis in mice, we assessed immune infiltration and epithelial damage in the colon by H&E staining. As shown in Figure 5A, treatment with rotenone showed a significant immune infiltration, thinning of the intestinal wall, and epithelial damage compared to that in the colon of CMC-Na+Saline group mice, which *L. plantarum* CCFM405 markedly restored. Similarly, the number of colonic cupped cells was reduced to half in the model group of mice after gavage with rotenone compared to that in the colon of healthy mice, whereas *L. plantarum* CCFM405 intake markedly increased their number. To further investigate whether the production of colonic inflammatory factors accompanied the intake of rotenone, we measured the concentrations of IL-1β, IL-6, and TNF-α in the colons of mice using ELISA. As shown in Figure 5D–F, the levels of IL-1β, IL-6, and TNF-α in the colons of mice in the rotenone-treated group were markedly increased compared to those in the colons of the normal healthy group mice (*p* < 0.05, *p* < 0.05 and *p* < 0.01). Compared with the model group, the colonic IL-6 and TNF-α levels were markedly lower in the CCFM405 intervention group of mice (*p* < 0.05, and *p* < 0.01), and IL-1β tended to decrease, but not significantly (*p* = 0.07). In addition, rotenone intake resulted in a significant decrease in relative mRNA expression of ZO-1 and Occludin protein (tight junction protein) in the mouse colon (*p* < 0.001 and *p* < 0.001), and supplementation with *L. plantarum* CCFM405 markedly upregulated the expression of these mRNAs (*p* < 0.05 and *p* < 0.05). Thus, these results suggest that *L. plantarum* CCFM405 markedly attenuates rotenone-induced histopathological features and inflammation in the colon.

### 3.6. Effects of L. plantarum CCFM405 on Gut Microbiota Diversity and Structure

**Figure 6:**
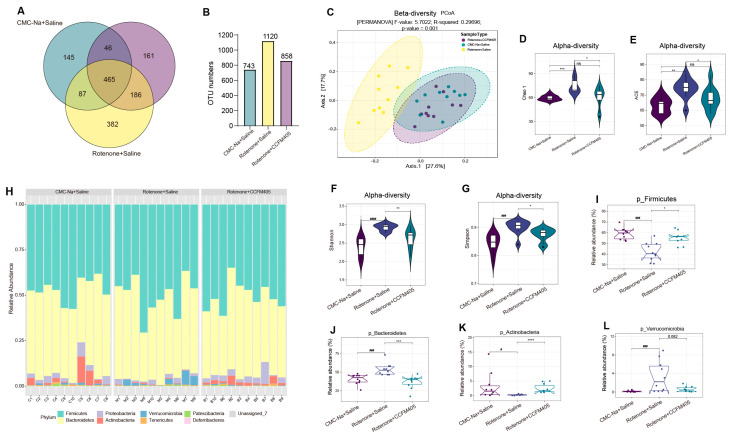
The effect of *L. plantarum* CCFM405 on gut microbiota diversity and its distribution at the phylum level. (**A**) A Venn diagram was utilized to illustrate the discrepancy in operational taxonomic units (OTUs) between the three groups; (**B**) Total number of OTUs in each group; (**C**) β-diversity measured by PCoA. PERMANOVA on the Bray–Curtis dissimilarity index: F-value = 5.7022, R^2^ = 0.29696, *p*-value = 0.001; (**D**–**G**) α-diversity indices reflected by the Chao1, ACE, Shannon, and Fisher index. Kruskal–Wallis tests calculated *p*-values; (**H**) the relative abundance of gut microbiota at the phylum level for each mouse is shown using stacked column bar graphs; (**I**–**L**) Mann–Whitney/Kruskal–Wallis tests/zero-inflated Gaussian fit model was used to conduct classical univariate statistical comparisons among selected phylum measured. Significance was considered for adjusted *p* (FDR, false discovery rate) < 0.05. *n* = 10/group. # *p* < 0.05, ### *p* < 0.001, #### *p* < 0.0001 signs Rotenone+Saline group vs. CMC-Na+Saline group; * *p* < 0.05, ** *p* < 0.01, *** *p* < 0.001, **** *p* < 0.0001 signs Rote-none+CCFM405 group vs. Rotenone+Saline group. “ns” means there was no noticeable difference between the groups.

We performed 16S rRNA amplicon paired-end sequencing to better understand how *L. plantarum* CCFM405 affects rotenone-induced intestinal microbiota dysregulation. After trimming and filtering, 4,604,467 excellent-quality reads were obtained from all fecal samples. Sequence similarities of at least 97% between these reads clustered them into 2721 OTUs. A Venn diagram was used to illustrate the number of common and unique OTUs across samples/groups. As shown in Figure 6A, 465 OTUs were shared by three groups, with 145, 382, and 161 OTUs unique to the CMC-Na+Saline, Rotenone+Saline, and Rotenone+CCFM405 groups, respectively. Notably, the number of OTUs found in these three groups were substantially different, at 743, 1120, and 858, respectively (Figure 6B). To estimate the β-diversity in gut microbiota and display the dissimilarity in bacterial composition among the three groups, a PCoA based on the Bray–Curtis dissimilarity index was performed. As shown in Figure 6C, an apparent cluster variation was observed among the three groups (PERMANOSIM, *p* = 0.002). PC1 and PC2 each contributed 27.6% and 17.7% of the overall variance in the data, respectively. Based on the Chao1, ACE, Shannon, and Simpson indices, within-sample α-diversity analysis showed a substantial increase in the richness of the gut microbial community following rotenone treatment compared to that in the CMC-Na+Saline group (*p* < 0.05) (Figure 6D–G). At the phylum level, the variations in gut microbiota structure between each group were analyzed, and the relative abundance was determined and displayed as a histogram of accumulation (Figure 6H). The most prominent phyla were Firmicutes, Bacteroidetes, and Proteobacteria. These three phyla were responsible for >90% of the microbial composition in all categories. Classical univariate statistical comparisons measured using the Mann–Whitney/Kruskal–Wallis tests/zero-inflated Gaussian fit model showed that the abundance of Firmicutes, Bacteroidetes, Acidobacteria, and Verrucomicrobia differed markedly between the CMC-Na+Saline and Rotenone+Saline groups (*p* < 0.05). Interestingly, *L. plantarum* CCFM405 treatment dramatically reversed this trend (Figure 6I–L).

### 3.7. Effect of L. plantarum CCFM405 on Rotenone-Induced Specific Differential Bacteria in PD Mice

**Figure 7:**
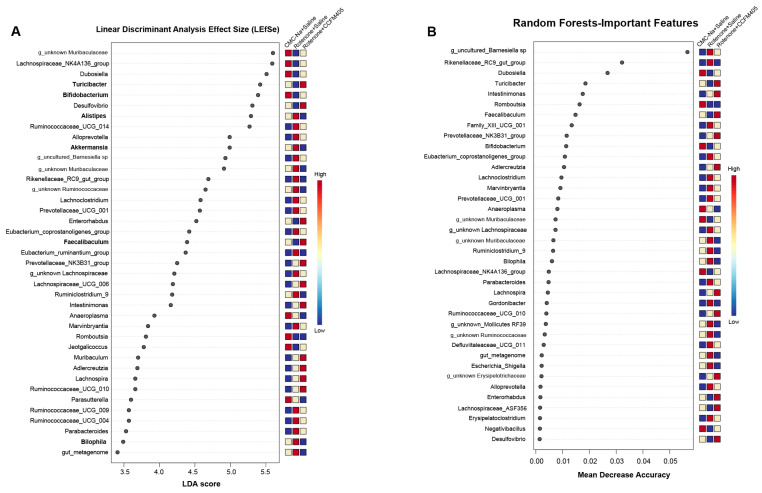
The general difference and determination of specific bacteria for discrimination among the three groups. (**A**) The linear discriminant analysis effect size (LEfSe) for three groups, with marked genera ranked in descending order by LDA value and a heatmap displaying matching genera that are either higher (red) or lower (blue); (**B**) the thirty-five most predictive genera were filtered by random forest classifier with feature importance scores. Feature importance was determined by calculating the mean decrease in model accuracy that resulted from randomly shuffling its values, *n* = 10/group.

A linear discriminant analysis (LDA) effect size (LEfSe) (log LDA score > 3) was performed to distinguish the specific differential bacteria among the three groups. As shown in Figure 7A, 40 taxa were markedly different and were ranked according to their LDA scores. Compared with the CMC-Na+Saline group, rotenone substantially reduced the abundance of Bifidobacterium, Faecalibaculum, and Turicibacter. Notably, treating mice with *L. plantarum* CCFM405 reversed this tendency and significantly promoted the growth of these three genera (Figure 7A). Mice treated with rotenone exhibited a markedly increased abundance of Alistipes, Akkermansia, Bilophila, Ruminococcaceae_UCG_004, and Ruminococcaceae_UCG_009 compared to those fed with *L. plantarum* CCFM405, which reversed this trend (Figure 7A). Random Forest analysis (RF) is a machine-supervised learning technique appropriate for high-dimensional data analysis. This method can categorize samples, distinguish biomarkers, and correct category errors by generating many decision trees [32]. The relevance of most of these top candidates in LEfSe was reinforced via RF classification (Figure 7B). The relative abundance of each of these 38 species is shown in Figure 7B (determined by the mean decrease in accuracy of their estimates). In particular, an increased abundance of Escherichia-Shigella was observed in the Rotenone+Saline group compared to that in the CMC-Na+Saline group (Figure 7B), and *L. plantarum* CCFM405 administration reversed this trend. The genera that primarily contributed to the differential abundance in the mice supplemented with *L. plantarum* CCFM405 were Bifidobacterium, Faecalibaculum, and Turicibacter, based on LEfSe and RF analyses.

### 3.8. Predicted Functional Changes in the Gut Microbiota

**Figure 8:**
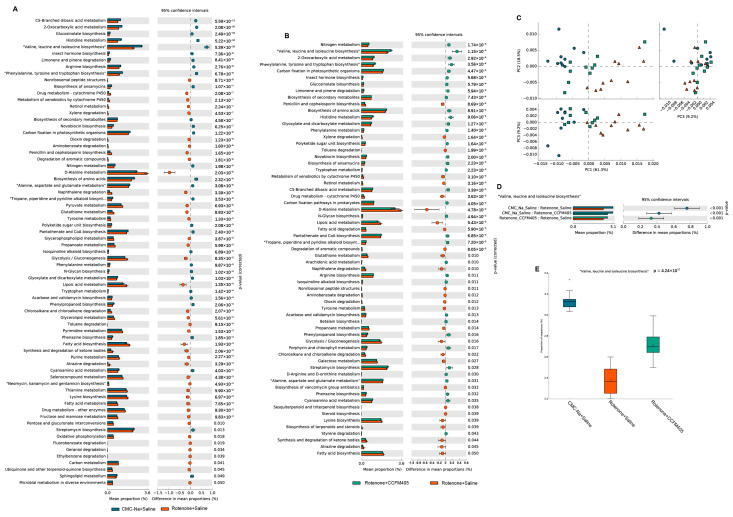
Functional prediction by Phylogenetic Investigation of Communities by Reconstruction of Unobserved States (PICRUSt 2). (**A**) Statistical analysis of difference of Kyoto Encyclopedia of Genes and Genomes (KEGG) pathways between CMC-Na+Saline group and Rotenone+Saline in levels II; (**B**) statistical analysis of difference of KEGG pathways between Rotenone+CCFM405 group and Rotenone+Saline in levels II; (**C**) PCA analysis of KEGG pathways among three groups; (**D**,**E**) statistical analysis of difference of “Valine, Leucine and isoleucine biosynthesis” pathways, *n* = 10/group.

The composition of the gut microbiota can affect its functional capacity. Thus, PICRUSt was performed to investigate the functional pathways associated with the gut microbiota composition changed by *L. plantarum* CCFM405 intake. As shown in Figure 8C, after principal component analysis (PCA) of KEGG pathways in the three groups of mice, we found their gut microbiota composition; those in the *L. plantarum* CCFM405-supplemented group were closer to those in the CMC-Na+Saline group, indicating that *L. plantarum* CCFM405 was beneficial in restoring the gut microbial imbalance after rotenone treatment. PC1, PC2, and PC3 accounted for 61.3%, 18.5%, and 9.2% of the total variance of the data, respectively. As shown in Figure 8A, compared with the CMC-Na+Saline group, the changes in the gut microbial function gene in the Rotenone+Saline group were mainly involved in amino acid metabolism-related pathways. These included branched-chain amino acid pathways (valine, leucine, and isoleucine biosynthesis), arginine biosynthesis, phenylalanine metabolism, and tyrosine and tryptophan biosynthesis, which were markedly downregulated. Interestingly, a recently published study demonstrated that the branched-chain amino acid synthesis pathway and related synthetic genes of the gut microbiota are also significantly downregulated in patients with PD, resulting in lower levels of branched-chain amino acids in the feces and serum [38]. As shown in Figure 8B, supplementation with *L. plantarum* CCFM405 significantly increased the branched-chain amino acid synthesis pathway and ranked second among all differential metabolic pathways. Statistical analysis, as shown in Figure 8D, further suggests that *L. plantarum* CCFM405 may modulate the gut microbiota compositions and promote branched-chain amino acid synthesis.

### 3.9. L. plantarum CCFM405 Altered the Fecal Metabolite Profiles in Rotenone-Induced PD Mice

**Figure 9:**
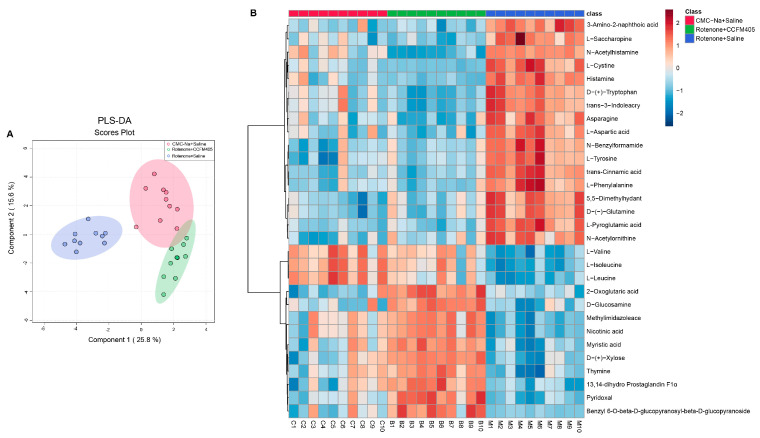
The PLS-DA model was utilized to analyze the compositions of fecal metabolites in each group of mice. (**A**) Two-dimensional score plot of PLS-DA of fecal metabolites among three groups; (**B**) cluster heat map of the top 30 differential metabolites ranked significant by one-way ANOVA for fecal metabolites among three groups, *n* = 10/group.

Growing evidence indicates that the gut microbiota influences the development and function of the brain. The gut–brain connection may be mediated by some microbial molecules produced in the gastrointestinal tract and can subsequently permeate the brain [39]. Therefore, we hypothesized that the potential mechanism of *L. plantarum* CCFM405 against PD could be related to improved fecal metabolite levels. A non-targeted metabolome based on liquid chromatography–mass spectrometry (LC–MS) was used to investigate the effects of *L. plantarum* CCFM405 on fecal metabolites. Scanning with LC–MS in positive (POS) and negative ion modes (NEG) identified 1007 and 445 metabolites, respectively. The compounds identified via NEG and POS were then integrated to screen for differential metabolites among groups. A cluster principle least squares discriminant analysis (PLS-DA) of all quality QC samples demonstrated that the results were stable and reliable (Figure S1). In addition, the PLS-DA score plots showed a clear separation among the three groups (Figure 9A). PCA analysis showed similar trends across the three groups (Figure S1). The top 35 fecal metabolites (FDR *p* < 0.05) among the three groups were screened using one-way ANOVA and Tukey’s honestly significant difference (HSD) post hoc test.

**Figure 10:**
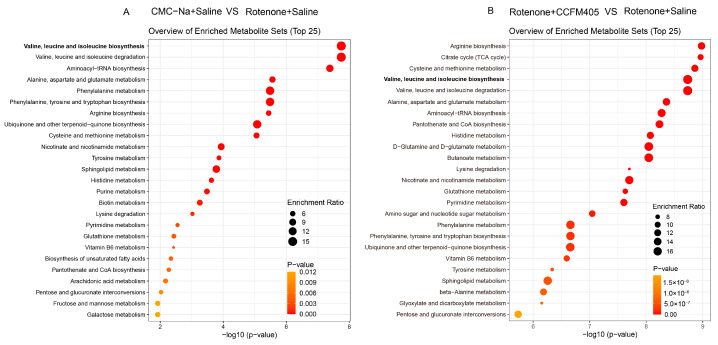
Metabolic pathway enrichment analysis of differential fecal metabolites. (**A**) Normal control versus PD group; (**B**) *L. plantarum* CCFM405 group versus PD group. The color of the histogram represents the metabolic enrichment pathways with significant differences. The size of the dots in the left panel means the enrichment rate, *n* = 10/group.

As shown in Figure 9B, some amino acids changed markedly among the three groups. Compared with the CMC-Na+Saline group, mice in the Rotenone+Saline group had higher levels of L-Cystine, Tryptophan, L-Tyrosine, L-Phenylalanine, and Glutamine, and *L. plantarum* CCFM405 supplementation was able to reverse this trend. In addition, the levels of L-Valine, L-Isoleucine, and L-Leucine in the feces of mice in the Rotenone+Saline group were markedly lower than those in the CMC-Na+Saline group. Interestingly, *L. plantarum* CCFM405 supplementation markedly increased the levels of these three amino acids in feces. Valine, isoleucine, and leucine, as essential amino acids, belong to branched-chain amino acids (BCAAs) and are often used in dietary supplementation. We further used orthogonal partial least squares discriminant analysis (OPLS-DA) to screen fecal differential metabolites of mice in the CMC-Na+Saline and PD groups, as well as those in the *L. plantarum* CCFM405 intervention and PD groups. We found that rotenone treatment could markedly reduce levels of lignoceric acid, valine, isoleucine, and leucine in feces (Figure S2 and Table S1). Compared with the Rotenone+Saline group, *L. plantarum* CCFM405 intake markedly increased the concentrations of nicotinic acid, L−valine, L−isoleucine, and L−leucine in feces (Figure S3 and Table S2). Moreover, based on the KEGG database, we performed pathway enrichment analysis of the screened differential metabolites (Figure 10A,B). Among the top 25 pathways, multiple amino acid metabolism pathways were markedly enriched in the CMC-Na+Saline and Rotenone+Saline groups, including valine, leucine, and isoleucine biosynthesis; alanine, aspartate, and glutamate metabolism; phenylalanine metabolism; phenylalanine, tyrosine, and tryptophan biosynthesis; arginine biosynthesis; and tyrosine metabolism (Figure 10A).

Notably, the results of the metabolic pathway enrichment analysis showed that the differential metabolic pathways between the Rotenone+CCFM405 and Rotenone+Saline groups also involved multiple amino acid metabolic pathways, with the highest enrichment ratios for arginine biosynthesis, followed by the citrate cycle (TCA cycle), and valine, leucine, and isoleucine biosynthesis (Figure 10B). The above results indicate that rotenone induced abnormal alterations in several amino acid metabolic pathways in the intestinal bacteria of PD mice, and these pathways were reduced compared to those in CMC-Na+Saline mice, especially regarding branched-chain amino acid biosynthesis. The supplementation with *L. plantarum* CCFM405 may exert a neuroprotective function by promoting the biosynthesis of branched-chain amino acids and by regulating intestinal microbiota composition.

### 3.10. L. plantarum CCFM405 Altered the Serum Metabolite Profiles of Rotenone-Induced PD Mice

**Figure 11:**
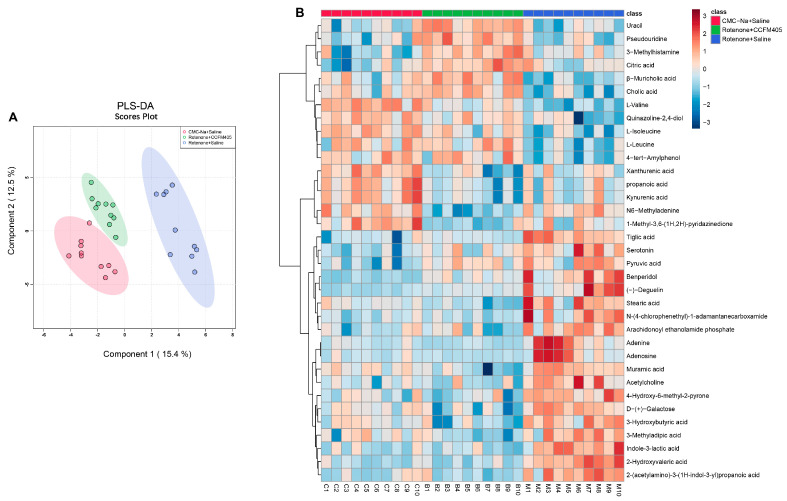
The PLS-DA model analyzed the composition of serum metabolites in each group of mice. (**A**) Two-dimensional score plot of serum metabolites PLS-DA in the three groups of mice; (**B**) cluster heat map of the top 30 differential metabolites ranked significant by one-way ANOVA in the three groups of mice, *n* = 10/group.

A recent multi-omics study revealed a strong link between the composition of the gut bacterial community, serum metabolic profile, and disease severity in patients with PD [40]. Therefore, we speculated that *L. plantarum* CCFM405 might affect serum metabolite composition in mice by modifying the intestinal microbiota composition and microbial metabolites. Scanning with LC-MS in POS and NEG identified 159 and 191 serum metabolites, respectively. The compounds identified via NEG and POS were then integrated to screen for differential metabolites among groups. A PLS-DA cluster analysis of all QC samples demonstrated that the results were stable and reliable (Figure S4). In addition, the PLS-DA score plots showed a clear separation among the three groups (Figure 11A). Similar trends were observed across the three groups in PCA (Figure S4). The top 35 serum metabolites (FDR *p* < 0.05) among the three groups were screened using one-way ANOVA and Tukey’s HSD post hoc test. As shown in Figure 11B, compared with the CMC-Na+Saline group, mice in the Rotenone+Saline group had lower levels of L-Valine, L-Isoleucine, and L-Leucine, and *L. plantarum* CCFM405 intake was able to reverse this trend.

**Figure 12:**
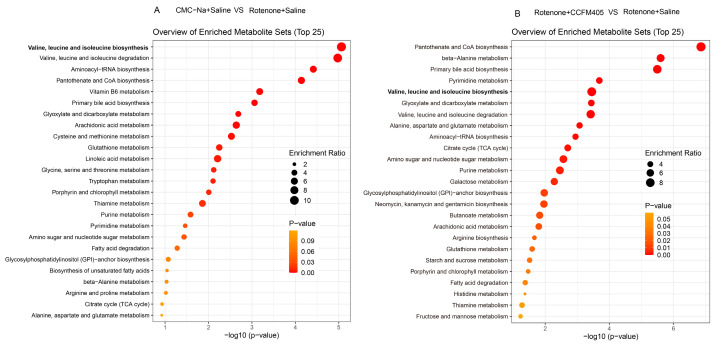
Metabolic pathway enrichment analysis of differential serum metabolites. (**A**) Normal control versus PD group; (**B**) *L. plantarum* CCFM405 group versus PD group. The color of the histogram represents the metabolic enrichment pathways with significant differences. The size of the dots in the left panel means the enrichment rate, *n* = 10/group.

We further used the OPLS-DA model to screen differential metabolites in serum between the control and PD groups. We found that rotenone treatment could markedly reduce the levels of cholic acid, β-Muricholic acid, L-Alanyl-L-proline, valine, isoleucine, and leucine in serum (Figure S5 and Table S3). Compared with the Rotenone+Saline group, *L. plantarum* CCFM405 intake markedly increased the concentrations of lignoceric acid, nervonic acid, chenodeoxycholic acid, cholic acid, β-Muricholic acid, L-Valine, L-Isoleucine, and L-Leucine in feces (Figure S6 and Table S4). The levels of lignoceric acid, L-Valine, L-Isoleucine, and L-Leucine were consistent with those in the feces. Moreover, based on the KEGG database, we performed pathway enrichment analysis of the screened differential metabolites (Figure 12A,B). Among the top 25 pathways, multiple biological metabolic pathways were markedly enriched in the CMC-Na+Saline and Rotenone+Saline groups, including biosynthesis of valine, leucine, isoleucine, aminoacyl−tRNA, pantothenate, CoA, and primary bile acid; and metabolism of linoleic acid, alanine, aspartate, and glutamate (Figure 12A). Notably, the results of the metabolic pathway enrichment analysis showed that the differential metabolic pathways between the Rotenone+CCFM405 and Rotenone+Saline groups also involved multiple similar metabolic pathways, with the highest enrichment ratios for pantothenate and CoA biosynthesis, primary bile acid biosynthesis, pyrimidine metabolism, and arginine biosynthesis (Figure 10B). Furthermore, the administration of rotenone resulted in a decrease in valine, leucine, and isoleucine biosynthesis in the intestinal bacteria, which in turn led to a reduction in fecal and serum branched-chain amino acids. *L. plantarum* CCFM405 supplementation was able to increase the branched-chain amino acid levels in feces and serum.

## 4. Discussion

Age is a substantial risk element for the onset and progression of various diseases, particularly neurodegenerative diseases such as PD. Because no pharmacological treatments have been established to maintain brain health or cure PD, it is of utmost significance to determine dietary interventions that prevent brain aging and can be readily adopted in humans. This study revealed that *L. plantarum* CCFM405 ameliorated rotenone-induced motor deficits via the gut–brain axis, as evidenced by comprehensive investigations of PD-like mouse movement performance, survival of dopaminergic neurons, neuroinflammation, neurotransmitter levels, colonic inflammation, gut microbiota composition, and metabolite profile in the serum and feces.

In most individuals suffering from PD, gastrointestinal dysfunction, including constipation and inflammatory bowel disease, occurs before motor symptoms [35,41]. Previous studies have shown that rotenone can induce DAergic-targeted neuronal toxicity in the enteric nervous system (ENS) of murine models, resulting in gastrointestinal (GI) nerve damage and pathology [24,25,26]. Intriguingly, Perez-Pardo et al. reported that rotenone causes GI dysfunction, intestinal inflammation, and intestinal barrier disruption before motor impairment [25]. This supports the Braak-staging hypothesis that the underlying mechanism of PD starts in the intestine and progresses to the brain [42,43]. We investigated the effects of *L. plantarum* CCFM405 on constipation in rotenone-induced PD mice, and found that *L. plantarum* CCFM405 increased fecal particle counts, fecal weight, and moisture content (Figure 2E–G). The effect of probiotics on constipation has not been investigated in MPTP-induced PD mice [20,22,23]. Therefore, the precise mechanism underlying the ameliorative effects of *L. plantarum* CCFM405 on constipation in PD requires further investigation. In addition, *L. plantarum* CCFM405 improved movement disorders in rotenone-challenged PD-like mice, as evidenced by four behavior evaluation tests (Figure 2). Similarly, *L. plantarum* PS128 and DP189 have also been found to have a positive ameliorative effect on motor dysfunction in MPTP-induced PD mice [20,22]. Mechanically, *L. plantarum* PS128 and DP189 were reported to relieve motor dysfunction by inhibiting oxidative stress and neuroinflammation in the SN and reshaping the composition of gut microbes. *L. plantarum* CCFM405 was reported to have a strong antioxidant capacity, with a hydroxyl radical scavenging rate of 32.02% [28]. Unfortunately, we were unable to investigate the effects of *L. plantarum* CCFM405 on oxidative stress in the SN in this study. Reducing oxidative stress, as one of the pathogenic mechanisms of PD, has emerged as one of numerous novel pharmacological therapies that may provide new avenues for PD treatment [5]. Rotenone can also induce oxidative stress in PD mice [44], and some dietary antioxidants have also been reported to exert neuroprotective effects through the antioxidant defense system [45]. Therefore, in future research, a comprehensive investigation on the effect of *L. plantarum* CCFM405 on oxidative stress in PD mice is needed. Recently, a systematic review of preclinical evidence also found that *L. plantarum* is the most reported in the treatment of PD [46], suggesting that *Lactobacillus plantarum* may have more potential than other probiotics for PD treatment. In future studies, comparative genomics analysis can be conducted on *L. plantarum* to find key core and functional genes in effective strains to provide guidance for screening out probiotics with therapeutic effects for PD.

Multiple investigations have established that intestinal microbiota imbalances emerge in patients with PD and PD-like animal models [47,48,49], implying that modifications in the intestinal microbiota have an essential role in the pathophysiology of PD. Rotenone treatment markedly altered the gut bacterial composition and increased gut microbiota diversity (Figure 6), which is consistent with the higher diversity of intestinal microbiota in patients with PD compared to that in healthy people [47,50]. Other PD-like rodent studies have also demonstrated a rise in the α-diversity of the gut microbiota [50]. Notably, *L. plantarum* CCFM405 reversed this tendency, which was consistent with alterations in the gut microbiota of mice fed CMC-Na+Saline. However, MPTP treatment markedly reduced gut microbiota diversity [22,51], which is inconsistent with the results of patients with PD. Herein, *L. plantarum* CCFM405 intake was observed to elevate the relative abundance of *Bifidobacterium*, *Turicibacter*, and *Faecalibaculum*, while reducing the relative abundance of *Alistipes*, *Bilophila*, *Akkermansia*, and *Escherichia-Shigella* (Figure 7). *Bifidobacteria*, which are widely used as probiotics, have been shown to have neuroprotective potential against neurological disorders in multiple animal models [52,53,54,55,56,57]. *Bifidobacterium animalis* consumption reduced several non-motor manifestations, including sleep quality, sadness, GI symptoms, and motor symptoms accompanied by alterations of gut microbiota and serum metabolites, according to newly published research [58]. In the current study, mice administered rotenone showed a markedly reduced abundance of the genus Faecalibaculum compared to that of the CMC-Na+Saline group mice. *Faecalibaculum*, as SCFA-producing bacteria, have been observed to be reduced in the intestines of aging mice [59]. A recent cohort study involving 96 patients with PD and 85 controls reported that the content of SCFA in feces was decreased in patients with PD. In addition, fecal SCFA levels were correlated with motor and cognitive function severity in patients with PD and with specific changes in pro-inflammatory bacteria [60]. Our data suggest that rotenone treatment decreased *Turicibacter* abundance and that *L. plantarum* CCFM405 markedly increased its relative abundance. A recent clinical study indicated a significant reduction in the abundance of the genus *Turicibacter* in patients with PD [61]. Conversely, two studies suggested that *Turicibacter* might also be a “potentially good bacterium” for metabolic diseases [62,63]. The abundance of pro-inflammatory *Bilophila* is markedly increased in patients with PD. Baldini et al. suggested that *Bilophila* may be a biomarker of PD progression [61], as it might affect human sulfur metabolism via its taurine-degrading capacities [64]. In addition, according to the findings of our study, rotenone treatment increased the abundance of *Bilophila*, and *L. plantarum* CCFM405 treatment markedly decreased its relative abundance. *Escherichia_Shigella* [65] and *Alistipes* [66] have been reported to have strong pro-inflammatory abilities that are significantly increased in patients with disease. Consistent with our results, the genera *Alistipes* were the major enriched components in patients with PD [67]. Likely, *Bifidobacterium breve* CCFM1067 reduced the abundance of *Escherichia-Shigella* [51].

Many studies have shown that individuals with PD have a much higher prevalence of *Akkermansia* and *Akkermansia muciniphila* than healthy participants, suggesting that *Akkermansia* may be associated with the pro-inflammatory state of PD [16]. Interestingly, in our study, rotenone intake markedly increased the abundance of *Akkermansia*, which is consistent with the results of patients with PD. However, MPTP treatment markedly reduced the abundance of *Akkermansia* [51]. Therefore, compared with MPTP-induced PD mice, rotenone may better simulate the intestinal microbial composition of patients with PD in terms of intestinal microbial diversity and some core intestinal bacteria. Furthermore, *L. plantarum* CCFM405 treatment also markedly decreased the relative abundance of *Akkermansia*, which adheres to the intestinal epithelium and is tightly connected to the mucosal layer. By promoting mucus production, it has been hypothesized that *Akkermansia* would increase the intestinal epithelium’s integrity [68,69]. Some investigations have shown a connection between increased *Akkermansia muciniphila* and intestinal permeability, probably via mucus degradation [70]. A recent study analyzing colon tissue samples from patients with PD found pathological manifestations, including barrier damage, mucosal inflammation, and immune activation in the colon tissue of patients with PD [26]. In our research, rotenone administration resulted in gut microbial dysbiosis, colonic inflammation, and downregulation of intestinal tight junction proteins with a rise in *Akkermansia*, and *L. plantarum* CCFM405 supplementation facilitated the reversal of these trends (Figure 5, Figure 6 and Figure 7).

We also found a marked decrease in the BCAAs biosynthesis; biosynthesis of amino acid, alanine, aspartate, and glutamate metabolism; arginine biosynthesis; and pantothenate and CoA biosynthesis pathways from the PICRUSt-predicted functional KEGG pathways in the Rotenone+Saline group (Figure 8A). This indicates that adverse changes in the gut microbiota function are a potential risk for PD, similar to previous studies [38,71]. Interestingly, we found that *L. plantarum* CCFM405 increased BCAAs and biosynthesis of amino acid pathways, indicating that the *L. plantarum* CCFM405-induced restructuring of the gut microbiota contributes to promoting the biosynthesis of amino acids, especially BCAAs. Subsequently, using a non-targeted metabolome investigation, we observed that mice exposed to rotenone had substantially reduced concentrations of BCAAs in their feces and serum. *L. plantarum* CCFM405 supplementation was able to upregulate BCAA levels in feces and serum, suggesting that it facilitates the biosynthesis of BCAAs by reconstituting the composition of the intestinal microbiota, and increasing the levels of BCAAs in the feces and serum of mice.

The levels of BCAAs and aromatic amino acids (AAAs) in plasma were markedly lower in patients with PD, and these levels were inversely correlated with Hoehn-Yahr scoring (disease severity) and strongly associated with the Desulfovibacteriaceae, Aminoacidococcaceae, and Erysipelaceae families in the gut microbiota, according to cohort research conducted in 106 patients with PD and 114 controls. Moreover, individuals with advanced PD exhibit a decreased abundance of essential genes involved in BCAA biosynthesis in their feces, and this leads to decreased BCAA concentrations in feces or reduced plasma BCAA levels [38], which is consistent with our results. Based on our data, we assume that the decrease in amino acid production by gut bacteria, which might decrease the proportion of amino acids absorbed, may be associated with their reduction in serum. While MPTP can induce alterations in gut microbial functions, especially increased LPS and peptidoglycan biosynthesis, *L. plantarum* PS128 could inhibit LPS and peptidoglycan biosynthesis [20], suggesting that *L. plantarum* CCFM405 and PS128 could affect brain function by regulating the function of gut microbes. In addition to probiotics, curcumin, as a functional food component, has been reported to exert a neuroprotective effect in MPTP-induced PD mice by modulating the gut microbiota along with key metabolites, such as tyrosine and dopa [72].

Gut bacteria are now known to be essential regulators of both health and illness. This regulator can affect the absorption and metabolism of nutrients consumed, which may have significant repercussions on the pathophysiology of the host [73]. Notably, gut microbes may help promote the biosynthesis and use of amino acids [74], which can then be absorbed by the intestine and stored in the blood [75]. Hence, the microbiota in the gut may affect the concentrations of plasma amino acids. Emerging evidence indicates that individuals with PD suffer from unbalanced gut microbiota compositions [16,76], which suggests that gut microbes are implicated in the pathophysiology of PD. The brain requires BCAAs as a nitrogen source to keep the glutamate–glutamine cycle between astrocytes and neurons functioning correctly [77]. By activating glutamate dehydrogenase, BCAAs can potentially accelerate the breakdown of glutamate [78]. Through a process known as transamination, BCAAs are converted into branched-chain keto acids, which contribute to a “buffering effect” against neurotoxic levels of glutamate [77]. It has been shown that elevated levels of glutamate are intimately linked to the onset and progression of PD owing to their excitotoxicity, oxidative stress, and immunological excitotoxicity [79]. Hence, by affecting glutamate metabolism, BCAAs may have a positive function in PD. Current findings have demonstrated that BCAAs have a growing crucial role in the brain, such as promoting neurotransmitter synthesis, participating in intracellular signaling, regulating levels of inflammation, and influencing mitochondrial function [80,81,82]. In addition, BCAAs can cross the blood–brain barrier and reach the central nervous system via large neutral amino acid transporters, thereby altering brain function and behavior [83].

Although our current findings revealed that *L. plantarum* CCFM405 restructured the gut microbiota composition, increased BCAAs levels in the mouse feces and serum, decreased colonic inflammation and neuroinflammation, protected dopaminergic neurons in the SN, and alleviated motor deficits, our study had some limitations. For example, because of the limited number of animals used in each group, we did not conduct an untargeted metabolomics analysis of the SN and striatum in mice to investigate how *L. plantarum* CCFM405 affected the metabolic profile of the mouse brain via the microbiota–gut–brain axis. Targeting oxidative stress and α-syn deposition has emerged as a therapy for PD. Unfortunately, we did not investigate whether *L. plantarum* CCFM405 can affect the change in the oxidative stress and α-syn deposition in rotenone-induced PD mice. In future studies, different biomarkers in PD should be assessed and comprehensive metabolomic analysis (feces, serum, and brain metabolites) in multiple PD mouse models (such as MPTP or 6-OHA-induced and Syn^Tg^ mice) should be conducted to clarify the mechanism of probiotics in alleviating PD.

## 5. Conclusions

Overall, this study indicates a novel and convincing association between the intestinal microbiota and motor function by BCAAs in PD mice fed *L. plantarum* CCFM405. *L. plantarum* CCFM405 restructured the gut microbiota composition, altered the microbiota-derived metabolite profile, increased BCAAs levels in mice feces and serum, decreased colonic inflammation and neuroinflammation, protected dopaminergic neurons in the SN, and alleviated motor deficits. The effect of *L. plantarum* CCFM405 on alterations in the microbiota–metabolite–brain axis should be further evaluated in human studies. Once validated, *L. plantarum* CCFM405 can be implemented as a novel intervention strategy for PD management.

## Data Availability

The data sets generated during and/or analyzed during the current study are either shown in the manuscript and Supplementary Material or are available from the corresponding author on reasonable request.

## Supplementary Materials

The following supporting information can be downloaded at https://www.mdpi.com/article/10.3390/nu15071737/s1, Figure S1. PCA analysis of fecal metabolites between groups in positive and negative ion mode; Figure S2. Differences in fecal metabolites between normal control mice and PD group mice; Figure S3. Differential fecal metabolites of mice in the *L. plantarum* CCFM405 group versus the PD group; Figure S4. PCA analysis of serum metabolites in three groups of mice in positive and negative ion mode; Figure S5. Differential serum metabolites between normal control mice and PD group mice; Figure S6. Differential serum metabolites of mice in the *L. plantarum* CCFM405 group versus the PD group; Table S1. The top 30 different fecal metabolites in FC value and P adjusted value between normal mice and rotenone-induced PD mice; Table S2. Top 30 different fecal metabolites in FC value and P adjusted value between the intervention group of *L. plantarum* CCFM405 and rotenone-induced PD mice; Table S3. The top 30 different serum metabolites in FC value and P adjusted value between normal mice and rotenone-induced PD mice; Table S4. Top 30 different serum metabolites in FC value and P adjusted value between the intervention group of *L. plantarum* CCFM405 and rotenone-induced PD mice; Table S5. Primers information used for qRT-PCR.
