# Supplementary material for: Lactobacillus plantarum CCFM405 against Rotenone-Induced Parkinson’s Disease Mice via Regulating Gut Microbiota and Branched-Chain Amino Acids Biosynthesis
Source: Nutrients. 2023 Apr 1;15(7):1737. doi: 10.3390/nu15071737 (PMC10096885; doi:10.3390/nu15071737)
Supplement: Supplementary file 1 [file nutrients-15-01737-s001.zip › nutrients-2297178-supplementary.pdf]

Supplementary Information

Supplementary methods

Behavioral Test

The **pole test** is a reliable method for evaluating bradykinesia in mouse PD models. A 0.5-meter-long, 1-centimeter-diameter, non-adhesive gauze-wrapped pole with a 2-centimeter-diameter, spherical protuberance was inserted for the pole descent test. Mice were placed on top of the pole with their heads facing down, and their drop back into their home cage was timed. Timing began when the animal was released by the researcher and concluded when one hind limb reached the bottom of the home cage. The test was administered three times to each animal, commencing the day after the last treatment, and the average of the three trials was computed for statistical analysis.

The **Balance beam walking test** was used to assess coordination and balance of movement. The apparatus was built on the basis of a previous study using 2 horizontal logs (5 mm square and 10 mm square). All mice were trained for 2 consecutive days (3 times a day on the largest size of balance beam) and tested for 3 days. The mice were placed at the open end of the walking log and trained to run towards a closed box. Mice that did not run into the box within 60 s were removed from the beam and those that accidentally fell off the walking beam were placed back in the cage for 2 min. A 5 min acclimatisation period was required before training and testing to obtain reliable results. The latency of each run and the number of times the hind limbs slipped out of the walking log during the run were recorded for subsequent analysis. On the day of testing, each mouse was tested 3 times (1 min between tests). The time taken to cross the beam into the end point was recorded until completion or a maximum of 60 s. If the mouse collapsed or turned back to the starting point in the middle of the run, the test was repeated. If the mouse was unable to walk to the end point in 60 s or consistently fell off the beam, the total time of 60 s was recorded. The average time was taken and the mean of the three trials was calculated for statistical analysis.

Motor balance was evaluated by using **Rota-rod test**. The RT-01 mouse rotarod (ZB-200, TECHMAN) equipped with automated timers and falling sensors was utilized

throughout both the training session and actual test. On day 20, all mice were trained at a rotational speed of 10 rpm for 180 seconds three times, proceeded by 20 rpm on day 21 and 30 rpm on day 22. On the day of the test, each mice was rotated at 30 rpm for 180 seconds three times (30-minute intervals between tests); the latency time to fall was documented, and the average time was calculated.

The open field test is widely used to evaluate the locomotion of animals. In a light behavioral testing environment, the OFT was administered in a 40 cm 40 cm 40 cm white box with a video camera placed immediately above the center. The mice were put in the box's center region for 10 minutes of spontaneous activity, and the screening area was separated into a central region and four corner regions. For the purpose of assessing the locomotor activity of mice in an unknown environment, an automated video-tracking system was utilized to record and evaluate the amount of time mice spent running, walking, and staying immobilized.

AB-PAS and HE staining of colon

Colon tissues (0.5 cm) were fixed in 4% (w/v) formalin solution, dehydrated in ethanol, embedded in paraffin, sliced, and stained with H&E. Goblet cell numbers were measured using AB-PAS staining.

Quantitative Real-Time Polymerase Chain Reaction (qRT-PCR)

Following the protocol described using total RNA extraction reagents (Vazyme, R401-01), RNA was extracted from the brain and intestinal tissue. Using the qPCR kit (Vazyme #R333, Nanjing, China), the extracted RNA was reverse-transcribed into cDNA. SYBR Green Supermix was used for quantitative PCR on a BioRad-CFX384 machine (Bio-Rad, California, USA). We used the cycle threshold (Ct) and normalized using the 2−ΔΔCt technique relative to Gapdh to measure the relative expression of genes. In the Supplementary Materials Table S5, the RT-qPCR primers were detailed.

Enzyme-Linked Immunosorbent Assay

Inflammatory cytokines (TNF-α, IL-β, IL-6, and IL-10) were measured using enzyme-linked immunosorbent assay (ELISA) kits according to the manufacturer's protocol (R&D Systems, Minnesota, USA). All experimental procedures were performed according to the manufacturer's instructions.

Metagenomic sequencing

Total genomic DNA was extracted from each stool samples (50 mg per sample) using a FastDNA® Spin Kit for Feces (MP Biomedicals, California, USA) according to the instructions provided by the manufacturer. Gel electrophoresis and spectrophotometry were utilized to examine DNA content and quality (Nanodrop 1000, NanoDrop Technologies, Wilmington, DE, USA). The V3-V4 region of the 16S rRNA gene was amplified by PCR using primers 341F (5′-CCTAYGGGRBGCASCAG-3′) and 806R (5′-GGACTACNNGGGTATCTAAT-3′) and Premix TaqTM (Takara, Dalian, China) according to the instructions. The amplification comprised of an initial denaturation stage at 95 °C for 5 minutes, followed by 30 cycles of 95 °C for 30 seconds, 52 °C for 30 seconds, and 72 °C for 30 seconds, and a final extension step at 72 °C for. After cycling, a final extension at 72 °C for 5 minutes was performed, and the reaction was then kept at 4 °C. The PCR products were subsequently purified using the DNA Gel/PCR purification miniprep test kit (Biomiga, BM-DC3511, China).

Preparation of fecal samples for untargeted metabolomics analysis

Fecal untargeted metabolomics was based on liquid chromatography-mass spectrometry (LC-MS). Fecal were stored at -80°C and thawed at 4°C before preparation. Eight times volume of solvent (methanol: acetonitrile: water = 2:2:1) were added to each fecal sample (50 mg) to extract metabolites. Magnetic beads were added to the fecal solvent and vortexed to mix (65 Hz, 6 times with an interval of 10 seconds). And then centrifugation at 4 °C at 14,000 rpm for 15 min. The supernatants were transferred to a new 1.5 ml centrifuge tube and dried in a centrifugal vacuum evaporator (SPD131DDA, Thermo Scientific, USA), re-dissolved with 200 μl of mixed solvent (methanol/water = 4:1), and then centrifuged at 4 °C at 14,000 rpm for 15 min.

Preparation of serum samples for untargeted metabolomics analysis

Before preparation, serum was frozen at -80 °C and thawed at 4 °C. To precipitate protein from blood samples, four times the volume of methanol was added, followed by vortexing for 30 seconds, ultrasonication at 4°C for 15 minutes, storage at -20°C for two hours, and centrifugation at 4°C for 15 minutes (15,000g, 4°C). The 80μl of supernatant was transferred to a fresh 1.5 ml Eppendorf tube and concentrated using a

centrifugal vacuum evaporator. Randomly, the re-dissolved concentration in 80% methanol was assayed in a random way. Quality control (QC) samples were prepared by pooling an equal volume of reconstitution from every serum sample and were analyzed after every 10 plasma samples. Monitoring the system's consistency and repeatability via QC samples. A blank sample (80% methanol) was used to remove the background and eliminate noise.

Liquid Chromatography (LC) and Mass Spectrometry (MS) System

Samples were analyzed for non-targeted metabolites using an UltiMate-3000 ultra-performance liquid chromatography (UPLC) system coupled with a high-resolution Q-Exactive mass spectrometer (Thermo Fisher Scientific). A Waters Acquity UPLC T3 column (2.1 × 100 mm, 1.8 μm) was used, with an operating temperature of 35 °C. In positive mode, mobile phase A was 0.1% formic acid and mobile phase B was 100% methanol; in negative mode, mobile phase A was 5 mM ammonium acetate and mobile phase B was 100% methanol. The gradient elution conditions were: 0–1.0 min, 2% B; 1.0–10 min, 2–98% B; 10–12 min, 98% B; 12–15 min, 2% B. The flow rate was maintained at 0.3 mL/min and the injection volume of both phases was 2 μL. The HESI source was operated under the following conditions: nitrogen flow of 35 and 15 arbitrary units for the sheath and auxiliary gas respectively, probe temperature and capillary temperature of 450 °C and 320 °C respectively and spray voltage of 3.8 and 2.8 kV in positive and negative mode respectively. For MS/MS spectra acquisition in later unknown structural annotation stages, the mass spectrometer was operated scanning for 50–1,070 Da in full-scan MS1 mode (full width at half height (FWHM) 70,000; automatic gain control (AGC) target 1e6; maximum injection time (IT) 100 ms) alternating with parallel reaction monitoring (PRM) MS/MS mode with normalized collision energy averaging 20, 40 and 60 eV (full width at half height 17 500; AGC target 5e4; max IT 50 ms; dynamic exclusion 10s).

Supplementary Figures and Tables

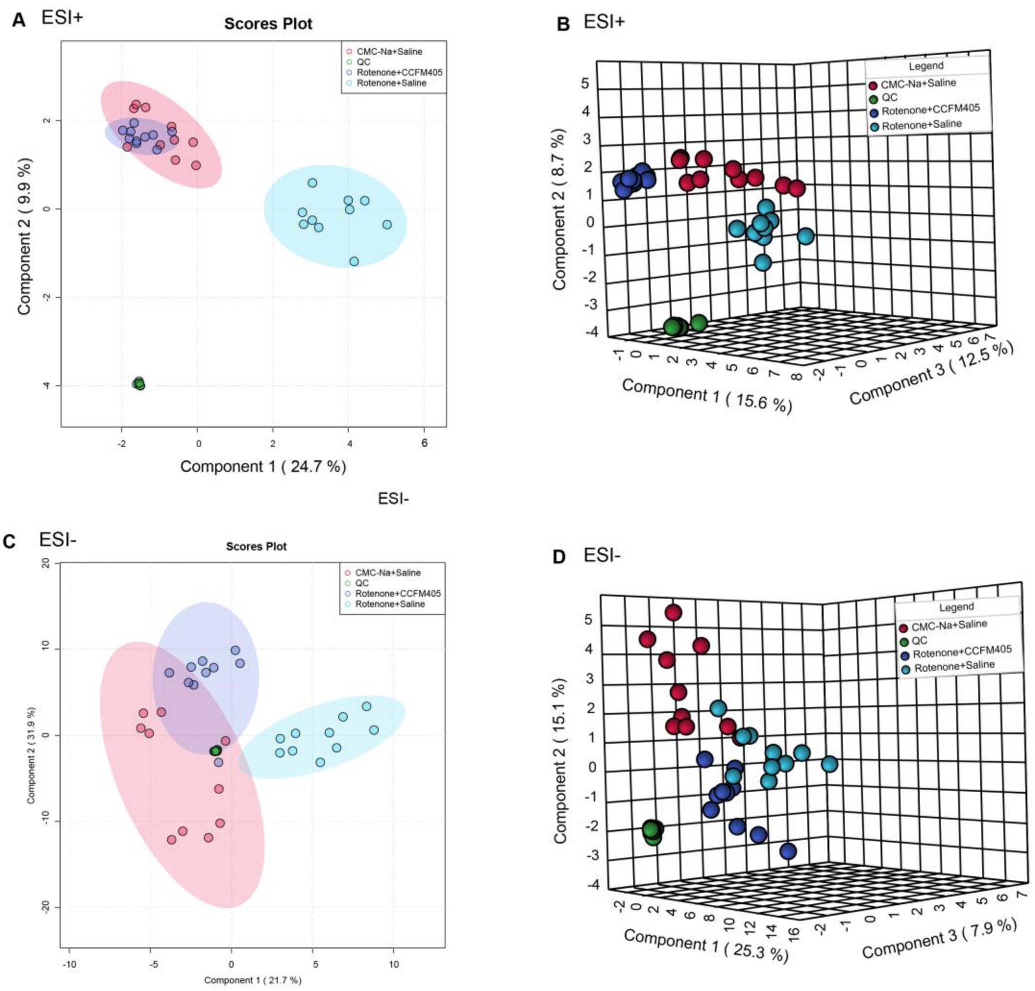

Figure S1. PCA analysis of fecal metabolites between groups in positive and negative ion mode. **(A)** Scores plot of 2D-PCA analysis of positive ion mode (ESI+); **(B)** Scores plot of 3D-PCA analysis of positive ion mode (ESI+); **(C)** Scores plot of 2D-PCA analysis of negative ion mode (ESI-); **(D)** Scores plot of 3D-PCA analysis of negative ion mode (ESI-).

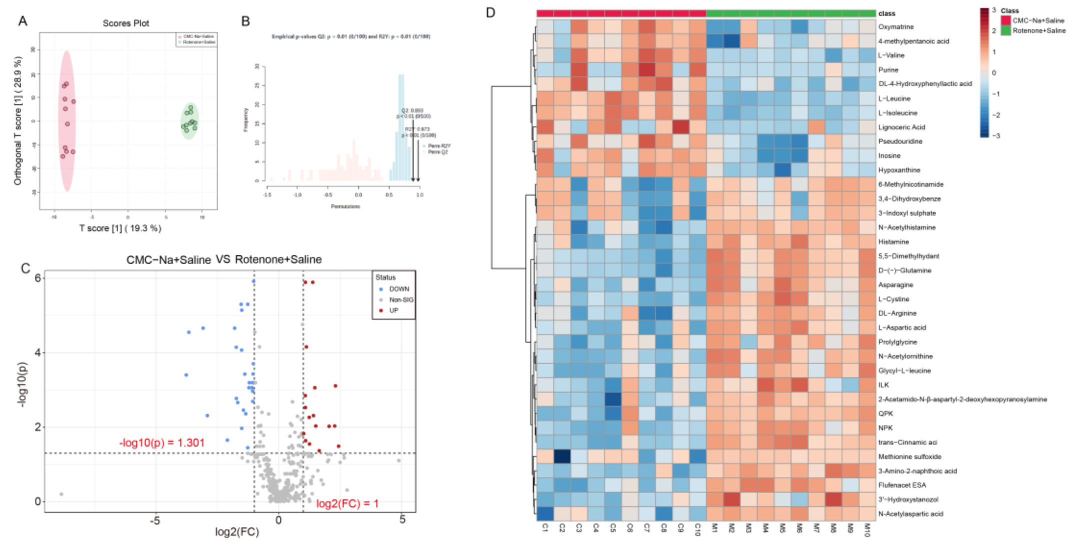

Figure S2. Differences in fecal metabolites between normal control mice and PD group mice. **(A)** Scores plot of OPLS-DA for normal control mice versus PD group; **(B)** Permutation testing with 100 iterations shows an excellent model fit (p < 0.01); **(C)** Volcano plot screening for significantly altered metabolites in normal control mice compared to PD group mice, with blue metabolism significantly down-regulated and red representing significantly up-regulated; **(D)** Heat map of clusters of differential metabolites between normal control mice and PD group mice with top 35 VIP values.

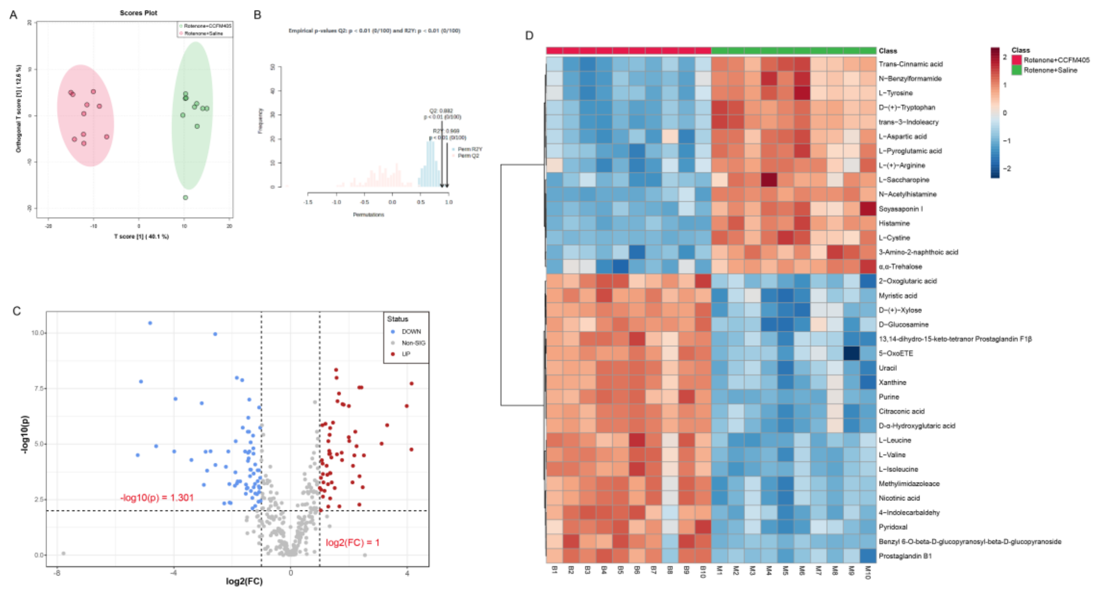

Figure S3. Differential fecal metabolites of mice in the L. plantarum CCFM405 group versus the PD group. **(A)** Scores plot of OPLS-DA for mice in the *L. plantarum*

CCFM405 group versus the PD group; **(B)** Permutation testing with 100 iterations shows an excellent model fit ($p < 0.01$); **(C)** Volcano plot screening for significantly altered metabolites in the *L. plantarum* CCFM405 group compared to mice in the PD group, with blue metabolism significantly down-regulated and red representing significantly up-regulated; **(D)** Cluster heat map of the top 35 differential metabolites in VIP values between mice in the *L. plantarum* CCFM405 group and mice in the PD group.

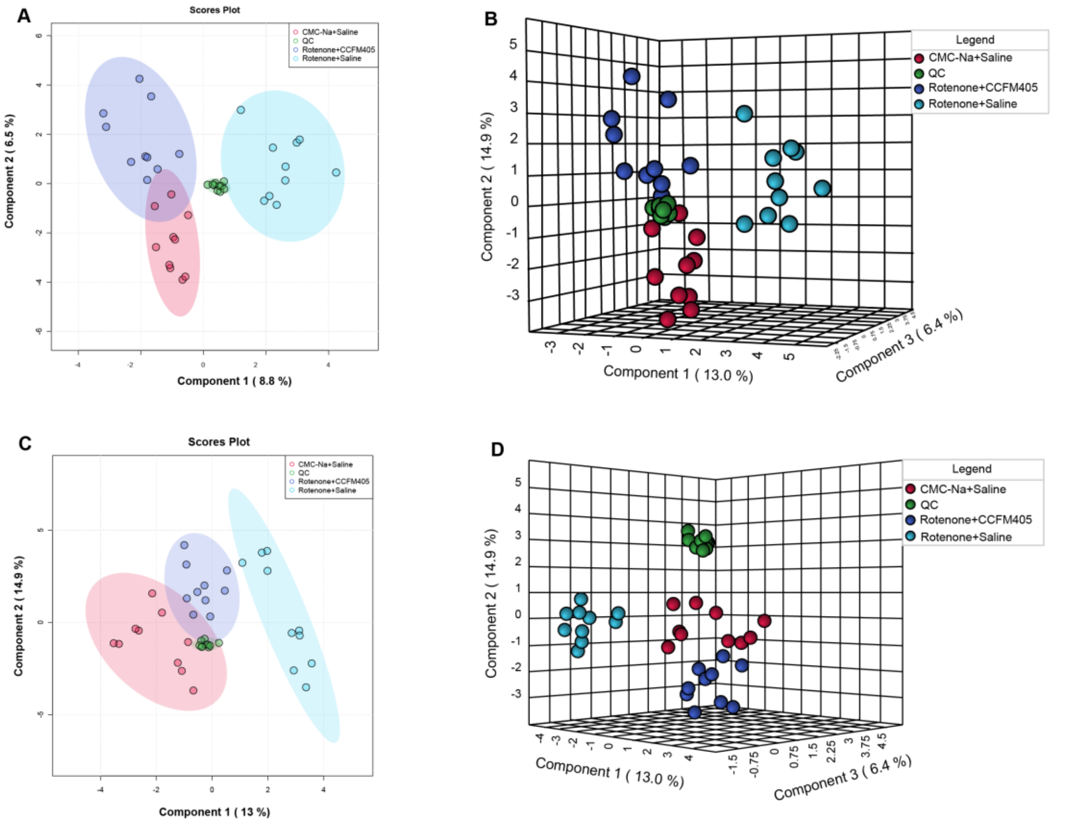

Figure S4. PCA analysis of serum metabolites in three groups of mice in positive and negative ion mode. **(A)** Scores plot of 2D-PCA analysis of positive ion mode (ESI+); **(B)** Scores plot of 3D-PCA analysis of positive ion mode (ESI+); **(C)** Scores plot of 2D-PCA analysis of negative ion mode (ESI-); (D) Scores plot of 3D-PCA analysis of negative ion mode (ESI-).

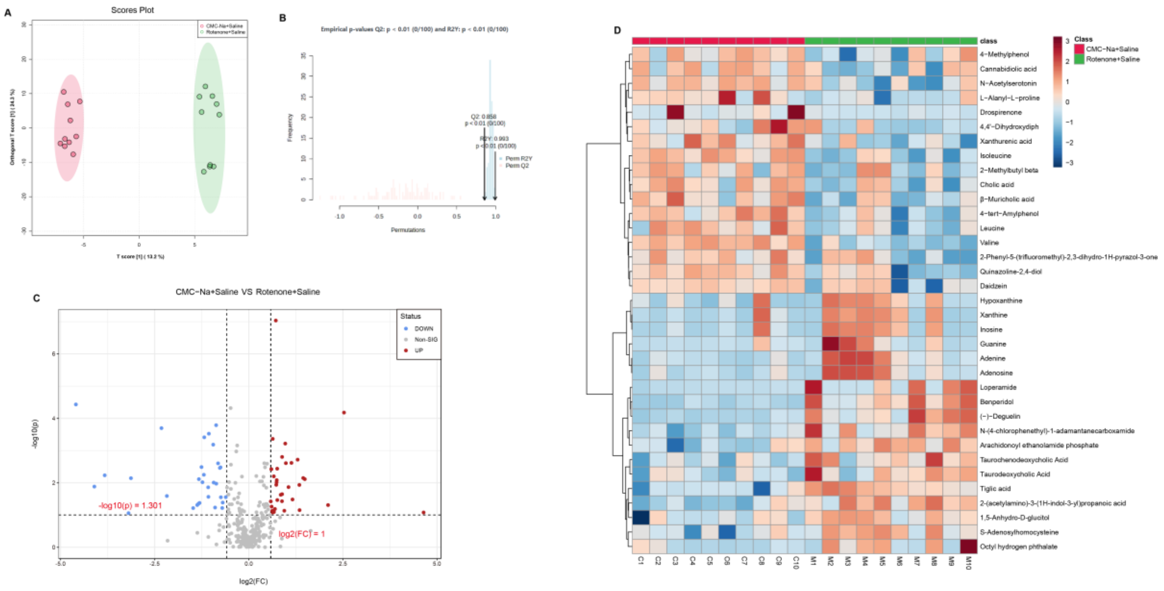

Figure S5. Differential serum metabolites between normal control mice and PD group mice. **(A)** Scores plot of of OPLS-DA scores for normal control mice vs. PD group; **(B)** Permutation testing with 100 iterations shows an excellent model fit (p < 0.01); **(C)** Volcano plot screening for significantly changed metabolites in normal control mice vs. PD group mice, with blue metabolism significantly down-regulated and red representing significantly up-regulated; **(D)** Cluster heat map of the top 35 differential metabolites in VIP values between mice in the normal control group and mice in the PD group.

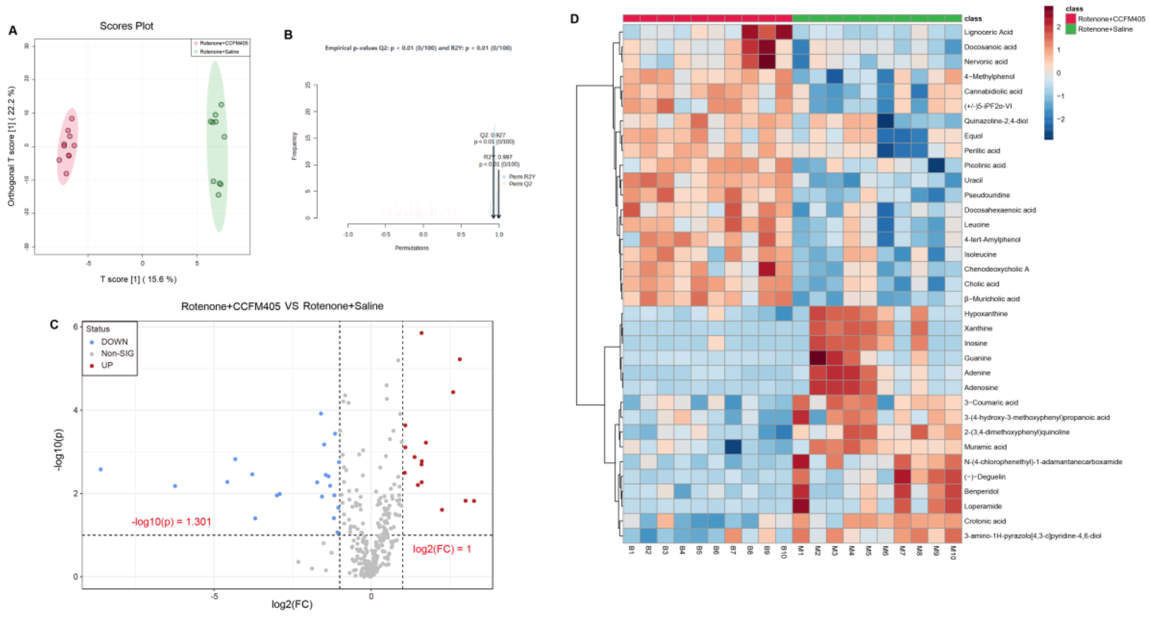

Figure S6. Differential serum metabolites of mice in the *L. plantarum* CCFM405 group versus the PD group. **(A)** Scores plot of OPLS-DA scores for mice in the *L. plantarum* CCFM405 group versus the PD group; **(B)** Permutation testing with 100 iterations shows an excellent model fit ($p < 0.01$); **(C)** Volcano plot screening for significantly altered metabolites in the *L. plantarum* CCFM405 group compared to mice in the PD group, with blue metabolism significantly down-regulated and red representing significantly up-regulated; (D) Cluster heat map of the top 35 differential serum metabolites in VIP values between mice in the *L. plantarum* CCFM405 group and mice in the PD group.

Table S1. The top 30 different fecal metabolites in FC value and P.ajusted value between normal mice and rotenone-induced PD mice.

Metabolite Name	FC	log2(FC)	P.ajusted	-log10(p)
L-Pyroglutamic acid	0.49183	-1.0238	1.21E-06	5.9179
L-Leucine	2.6093	1.3837	1.28E-06	5.8913
L-Isoleucine	2.1237	1.0866	1.28E-06	5.8913
5,5-Dimethylhydantoin	0.34481	-1.5361	5.00E-06	5.3011
trans-Cinnamic acid	0.41599	-1.2654	5.00E-06	5.3011
D-(-)-Glutamine	0.35127	-1.5094	7.20E-06	5.1424
3-Amino-2-naphthoic acid	0.11784	-3.0851	2.19E-05	4.6596
N-Acetylornithine	0.28727	-1.7995	2.19E-05	4.6596
L-Cystine	0.078737	-3.6668	2.82E-05	4.55
Adenosine	2.1807	1.1248	6.97E-05	4.157
Histamine	0.30054	-1.7344	7.13E-05	4.1471
Flufenacet ESA	0.35001	-1.5146	8.49E-05	4.0708
L-Tyrosine	0.48751	-1.0365	1.98E-04	3.7029
L-Saccharopine	0.48362	-1.048	3.73E-04	3.4288
NPK	0.38266	-1.3859	3.75E-04	3.4256
Asparagine	0.073124	-3.7735	3.96E-04	3.4022
L-Aspartic acid	0.43484	-1.2015	6.38E-04	3.1953
SPK	0.47622	-1.0703	6.38E-04	3.1953
D-(+)-Tryptophan	0.49386	-1.0178	6.38E-04	3.1953
butanoic acid	4.9584	2.3099	7.76E-04	3.11
DL-4-Hydroxyphenyllactic acid	2.7679	1.4688	8.71E-04	3.06
N-Acetylaspartic acid	0.42997	-1.2177	8.71E-04	3.06
QPK	0.46795	-1.0956	8.71E-04	3.06
Tyrosylalanine	0.47797	-1.065	0.001078	2.9674
7-Methylguanine	2.1149	1.0806	0.001417	2.8486
Glycyl-L-leucine	0.30132	-1.7307	0.0016874	2.7728
L-Serine	0.47942	-1.0606	0.0020263	2.6933
N-Acetylhistamine	0.31322	-1.6747	0.0021799	2.6616
2'-Deoxyadenosine	2.1211	1.0848	0.0029634	2.5282
2-Acetamido-N-β-aspartyl-2-deoxyhexopyranosylamine	0.36786	-1.4428	0.0034817	2.4582

Table S2. Top 30 different fecal metabolites in FC value and P.ajusted value between the intervention group of *L.plantarum* CCFM405 and rotenone-induced PD mice.

Metabolite Name	FC	log2(FC)	P.ajusted	-log10(p)
N-Acetylhistamine	0.035554	-4.8138	3.53E-11	10.452
Histamine	0.16703	-2.5819	1.11E-10	9.9545
L-Valine	2.9552	1.5632	4.54E-09	8.3433
D-(+)-Tryptophan	0.27909	-1.8412	1.03E-08	7.9857
L-Isoleucine	2.9873	1.5788	1.03E-08	7.9857
trans-3-Indoleacrylic acid	0.31693	-1.6578	1.32E-08	7.8806
L-Cystine	0.028615	-5.1271	1.53E-08	7.8162
Purine	17.781	4.1522	1.87E-08	7.7272
D-α-Hydroxyglutaric acid	5.3965	2.432	2.81E-08	7.5512
Citraconic acid	5.1324	2.3596	2.81E-08	7.5512
Uracil	3.164	1.6617	5.27E-08	7.2778
3-Amino-2-naphthoic acid	0.064992	-3.9436	9.18E-08	7.0371
trans-Cinnamic acid	0.37525	-1.4141	9.88E-08	7.0051
L-Leucine	3.0528	1.6101	1.17E-07	6.9334
Soyasaponin I	0.12127	-3.0437	1.44E-07	6.8414
D-(+)-Xylose	3.4375	1.7814	1.58E-07	6.8007
Xanthine	3.5353	1.8219	1.68E-07	6.7742
2-Oxoglutaric acid	4.0029	2.001	1.93E-07	6.7155
13,14-dihydro-15-keto-tetranor Prostaglandin F1β	15.83	3.9846	1.93E-07	6.7154
L-Pyroglutamic acid	0.4742	-1.0764	2.24E-07	6.6489
L-Saccharopine	0.3663	-1.4489	6.48E-07	6.1883
4-Indolecarbaldehyde	2.7444	1.4565	1.08E-06	5.9675
Pyridoxal	2.2931	1.1973	1.22E-06	5.9136
Benzyl 6-O-beta-D-glucopyranosyl-beta-D-glucopyranoside	9.9413	3.3134	1.40E-06	5.8532
Myristic acid	2.1336	1.0933	1.40E-06	5.8532
L-Aspartic acid	0.32103	-1.6392	1.82E-06	5.7407
L-Tyrosine	0.48667	-1.039	1.82E-06	5.7407
5-OxoETE	2.5637	1.3582	1.88E-06	5.725
D-Glucosamine	4.6622	2.221	2.77E-06	5.5579
α,α-Trehalose	0.36206	-1.4657	2.77E-06	5.5579

Table S3. The top 30 different serum metabolites in FC value and P.ajusted value between normal mice and rotenone-induced PD mice.

Metabolite Name	FC	log2(FC)	P.ajusted	-log10(p)
Valine	1.6449	0.71804	9.21E-08	7.0359
Benperidol	0.041487	-4.5912	3.70E-05	4.4322
Isoleucine	5.776	2.5301	6.62E-05	4.1795
2-(acetylamino)-3-(1H-indol-3-yl) propanoic acid	0.54848	-0.8665	1.63E-04	3.7877
(-)-Deguelin	0.20046	-2.3186	2.01E-04	3.6964
Tiglic acid	0.47981	-1.0595	2.99E-04	3.5247
N-(4-chlorophenethyl)-1-adamantanecarboxamide	0.44077	-1.1819	3.87E-04	3.4122
4-Pyridoxic acid	1.5549	0.63685	4.33E-04	3.3638
4-tert-Amylphenol	1.9581	0.96947	6.06E-04	3.2177
Arachidonoyl ethanolamide phosphate	0.51969	-0.94427	6.54E-04	3.1844
Xanthurenic acid	1.847	0.88521	0.0015728	2.8033
Quinazoline-2,4-diol	2.4594	1.2983	0.0019139	2.7181
N-Acetylserotonin	2.1938	1.1334	0.0024088	2.6182
Leucine	1.98	0.98547	0.0024568	2.6096
Indole-3-lactic acid	0.5683	-0.81527	0.0024893	2.6039
Taurochenodeoxycholic Acid	0.41889	-1.2554	0.003243	2.4891
4-Hydroxy-6-methyl-2-pyrone	0.59469	-0.74979	0.0033079	2.4804
1-(2-furyl) pentane-1,4-dione	0.58771	-0.76683	0.0034621	2.4607
Prostaglandin H2	1.6878	0.75511	0.0036418	2.4387
3-(2-Oxo-2,3-dihydro-1,3-benzoxazol-3-yl) propanoic acid	1.515	0.59934	0.0037747	2.4231
Kynurenic acid	1.515	0.59934	0.0037747	2.4231
S-Adenosylhomocysteine	0.43748	-1.1927	0.0056312	2.2494
Adenine	0.070507	-3.8261	0.0058516	2.2327
Glycine	1.5852	0.66463	0.0062989	2.2007
β-Muricholic acid	2.7246	1.446	0.0071089	2.1482
Loperamide	0.1143	-3.1291	0.0071837	2.1437
Cholic acid	2.8053	1.4882	0.007692	2.114
Taurochenodeoxycholic acid	0.39932	-1.3244	0.0076982	2.1136
3-Ureidopropionic acid	1.6733	0.74266	0.0082697	2.0825
3-(3,4-dihydroxyphenyl) propanoic acid	0.42628	-1.2301	0.0095898	2.0182

Table. S4 Top 30 different serum metabolites in FC value and P.ajusted value between the intervention group of *L.plantarum* CCFM405 and rotenone-induced PD mice.

Metabolite Name	FC	log2(FC)	P.ajusted	-log10(p)
Uracil	3.0381	1.6032	1.40E-06	5.853
β-Muricholic acid	7.0673	2.8212	6.01E-06	5.2214
Cholic acid	6.0971	2.6081	3.69E-05	4.4327
2-(3,4-dimethoxyphenyl) quinoline	0.33079	-1.596	1.21E-04	3.9187
Leucine	2.1222	1.0856	2.31E-04	3.636
N-(4-chlorophenethyl)-1-adamantanecarboxamide	0.45088	-1.1492	3.67E-04	3.4355
Chenodeoxycholic Acid	3.3449	1.742	6.02E-04	3.2207
Muramic acid	0.35441	-1.4965	6.67E-04	3.1761
4-tert-Amylphenol	2.1243	1.087	7.82E-04	3.107
Docosahexaenoic acid ethyl ester	2.6054	1.3815	0.0013333	2.8751
Adenine	0.04992	-4.3243	0.0014994	2.8241
Cannabidiolic acid	3.0553	1.6113	0.0016814	2.7743
3-amino-1H-pyrazolo[4,3-c] pyridine-4,6-diol	0.49283	-1.0208	0.0017647	2.7533
4-Methylphenol	3.0365	1.6024	0.0020048	2.6979
Xanthine	0.0025609	-8.6091	0.0026381	2.5787
D-Mannose 6-phosphate	2.097	1.0683	0.0031933	2.4958
Hypoxanthine	0.072577	-3.7843	0.0034585	2.4611
3-Coumaric acid	0.36735	-1.4448	0.0035944	2.4444
3-(3,4-dihydroxyphenyl) propanoic acid	0.39044	-1.3568	0.0038818	2.411
Adenosine	0.041922	-4.5762	0.0052915	2.2764
Isoleucine	3.0473	1.6075	0.0053669	2.2703
(-)-Deguelin	0.30377	-1.7189	0.0054063	2.2671
(+/-)5-iPF2α-VI	2.8019	1.4864	0.0062651	2.2031
13(S)-HpOTrE	0.40457	-1.3055	0.0065315	2.185
Inosine	0.013233	-6.2397	0.0066144	2.1795
Benperidol	0.13287	-2.9119	0.010283	1.9879
Taurochenodeoxycholic acid	0.44399	-1.1714	0.011089	1.9551
Loperamide	0.12504	-2.9996	0.011151	1.9527
3-(4-hydroxy-3-methoxyphenyl) propanoic acid	0.33823	-1.5639	0.011879	1.9252
Lignoceric Acid	8.0233	3.0042	0.014982	1.8244

Table. S5 Primers information used for qRT-PCR

Primer	Sequence (5'-3')	Location	Amplicon Size (bp)
ZO-1	GGGAGGGTCAAATGAAGACA	6366-6385	145
	GGCATTCCTGCTGGTTACAT	6510-6491	
Occluldin	ATAATGGGAGTGAACCCGACG	724-744	187
	CCACGATAATCATGAACCCCA	910-890	
Claudin-1	TGGATGGCTGTCATTGGGG	562-580	127
	ACCTGGCATTGATGGGGGT	688-670	
Gapdh	ACCACAGTCCATGCCATCAC	946-965	452
	TCCACCACCCTGTTGCTGTA	1397-1378	
